# Prediction and classification in equation-free collective motion dynamics

**DOI:** 10.1371/journal.pcbi.1006545

**Published:** 2018-11-05

**Authors:** Keisuke Fujii, Takeshi Kawasaki, Yuki Inaba, Yoshinobu Kawahara

**Affiliations:** 1 Center for Advanced Intelligence Project, Institute of Physical and Chemical Research (RIKEN), Osaka, JAPAN; 2 Department of Physics, Nagoya University, Nagoya, Aichi, JAPAN; 3 Japan Institute of Sports Sciences, Tokyo, JAPAN; 4 The Institute of Scientific and Industrial Research, Osaka University, Osaka, JAPAN; Waseda University, JAPAN

## Abstract

Modeling the complex collective behavior is a challenging issue in several material and life sciences. The collective motion has been usually modeled by simple interaction rules and explained by global statistics. However, it remains difficult to bridge the gap between the dynamic properties of the complex interaction and the emerging group-level functions. Here we introduce decomposition methods to directly extract and classify the latent global dynamics of nonlinear dynamical systems in an equation-free manner, even including complex interaction in few data dimensions. We first verified that the basic decomposition method can extract and discriminate the dynamics of a well-known rule-based fish-schooling (or bird-flocking) model. The method extracted different temporal frequency modes with spatial interaction coherence among three distinct emergent motions, whereas these wave properties in multiple spatiotemporal scales showed similar dispersion relations. Second, we extended the basic method to map high-dimensional feature space for application to actual small-dimensional systems complexly changing the interaction rules. Using group sports human data, we classified the dynamics and predicted the group objective achievement. Our methods have a potential for classifying collective motions in various domains which obey in non-trivial dominance law known as active matters.

## Author summary

Modeling complex collective motions is a challenging problem such as in biology, physics, and human behavior because the rules governing the motion are sometimes unclear. Then, researchers have usually used simple interaction model and explain global statistics. However, it remains difficult to bridge the gap between the dynamic properties of the complex interaction and the group-level functions. This study develops an effective framework to extract the dynamics of collective motion from data by data-driven modeling. Compared with conventional methods, our method can be applied to cases with the small numbers of group members or transient and complex changes of the behavioral rules. Our methods successfully discriminated group movements of well-known fish-schooling models and predicted the achievement of a group objective from actual basketball players’ position data. Our methods have a potential for outcome prediction and classification for various unsolved and complex collective motions such as in biology and physics.

## Introduction

Cohesive and attractive motion in groups of social organisms emerges from dynamic individual interactions. Collective motion has been understood mainly via microscopic modeling of individual behavior and macroscopic group statistics, which has attracted attention in broad research areas such as in biology [1–3], physics [4–6], and human behavior [7–9]. Most of researches elucidated that complex group motion can emerge from simple individual interaction rules [2, 3, 6] and shows a nonlinear change in global variables [2, 8] or statistics [1, 10], including phase transitions [6]. However, the relationship between the dynamic properties of the emerging complex interaction and the group-level functions has been unknown. Generally, the problem has been well-known as an active matter or non-equilibrium many-body system problem [11], in which some physical laws such as fluctuation-dissipation theorem [5] do not hold owing to the external or self-propelled force. Fundamental questions also remain about the bridge between individual and functional collective motion in applied contexts e.g., in human behavior [7–9], which has been also simply modeled as artificial multi-agents [12] and as “social force” in non-reciprocal interaction [7, 13, 14]. However, the modeling has been sometimes too complicated owing to its higher-level sociality [9, 15]. The collective motion problem requires a way to understand them in a data-driven and equation-free manner [16], because the standard parametric approach assumes that individual and statistical models are basically correct and sometimes lacks the flexibility to describe complex dynamics that occur in nature [17].

Decomposition methods of extracting modes from data about complex dynamical interactions of materials have been developed mainly in the field of fluid physics, including proper orthogonal decomposition (POD) [18, 19] or dynamic mode decomposition (DMD) [20, 21]. POD is a statistical decomposition that does not account for latent dynamics in data. In contrast, DMD can extract a mode that can yield direct information about nonlinear latent dynamics by assuming a discrete-time nonlinear dynamical system:

$$x_{t+1}=f(x_{t}),$$ (1)

where ***x***_*i*_ is a state vector on the state space M (i.e., $x\in M\subset R^{d}$) and ***f*** is a state transition function that assumes the dynamical system to be nonlinear. DMD originated in the fluid physics community as a method to decompose large-dimensional complex flows into a simple spectral representation based on coherent spatiotemporal structures [21]. For example, DMD has been used to extract the low-frequency mode in the fluid vortex along a wall, which was difficult with POD [20]. DMD has gone to be applied to data obtained from many complex phenomena [22] such as epidemiology [23] and neural activity [24]. Also in collective motion emerging from local interactions, various frequency modes will occur in the data, such as a low frequency mode in coordinative motion and a high frequency mode in competitive motion against other agents or the environment. Thus, we assumed that the dynamic modes can be extracted even from local dynamic properties of collective motion. Compared with conventional data-driven approaches such as nonlinear Laplacian spectral analysis [25] and sparse identification of nonlinear dynamical systems [16], DMD can yield direct information concerning dynamics without requiring explicit prior knowledge.

In general terms of the spatiotemporal behavior of many-particle motions, for example in material physics, dynamic structure factors at multiple spatiotemporal scales has been computed for various materials [26–28]. Recently, dynamic structure factors have been examined in active matter such as swimming particles [29] to reveal the spatiotemporal wave properties of the collective motion, such as whether the motion mimics that of a viscous fluid, an elastic solid, or a viscoelastic fluid. Building upon these researches, we examined the local and global dynamic properties of collective motion in terms of material or fluid physics using DMD and dynamic structure factor analysis.

Therefore, we first verify the validity of our approach using simple and well-known collective motion models [4, 6] to generate multiple distinct group behavioral patterns. For example, individual-based models that simulate swarming, torus-like, or parallel group behaviors of bird-flocking and fish-schooling [2] are good examples because the relationship between the properties of the local system and the emergence of global behavior is well-known and explicit. However, the contribution of the dynamic properties as a result of the simple interaction to the global emergent behaviors has been unknown. We thus perform DMD analysis to extract the local and global dynamic property of three model systems, and calculate the dynamic structure factor to investigate global dynamic properties of the system and their relationship with the DMD results.

However, the original DMD method has two main shortcomings when applied to the actual complex collective motion. One is that we need to have large data dimensions to extract the spatiotemporal coherent structure. This means that the number of decomposable modes and the expressiveness of the data will decrease when the group includes a small number of agents. The other shortcoming is that the coefficients of the decomposed modes are assumed to remain constant within the data interval (i.e., the manner of attenuation and vibration is consistent), so the expressiveness will also decrease for data from actual living systems, especially of humans, who flexibly change the rules of behavior according to the situation. A recent development is the use of Koopman spectral analysis with reproducing kernels [30], which is a generalization of the DMD framework. The new method can decompose the mode infinite functional space and then can acquire high expressiveness even for the time-varying interaction mode and in low-dimensional sequences through spectral decomposition of the Koopman operator [31]. We previously developed the classification and prediction methods further using the DMD spectrum and referred to the method as Koopman spectral kernels [32].

More complicated collective motions that emerge from more complex rules are one of the desirable applications for exploiting the advantages of this equation-free method. Organized human tasks such as navigation [33] or ballgame teams [9] provide excellent examples of complex dynamics and pose challenges in many research fields because of their switching and overlapping hierarchical subsystems [9], which are often characterized by recursive shared intentionality [15]. Previous work on data from a ballgame performed unsupervised classification of offensive team movements [34, 35]. However, these models did not fully represent the time-varying and interactions with defensive teams, and modeling methods robust enough to reflect individual relationships with group objectives (e.g., scoring) have not yet been developed. Generally, in human groups, supervisors (e.g., coaches or teachers) analyze group motions and agents (e.g., players or students) repeatedly practice moves that increase the probability of achieving group objectives. However, the selection of group strategies to achieve the goal might be a difficult problem, and thus requires the supervisors’ implicit experience-based knowledge. Previously, we reported that in a set of a ballgame data, three maximum attacker-defender distances separately predicted whether a team would score or not [9], but the study addressed only the outcome of a play, rather than its temporal evolution and the interactions that took place. Therefore, in this study, we map the latent dynamic characteristics of multiple players’ interactions in a ballgame to a feature space acquired by DMD with reproducing kernels. Our method allows us to classify the complex collective motion and predict the achievement of the group objective.

### Theoretical framework

#### Physical property extracted by basic dynamic mode decomposition

The decomposition method for analyzing dynamical systems is a popular approach that aims at extracting low-dimensional dynamics from data. Common techniques include global eigenmodes for linearized dynamics, discrete Fourier transforms, and POD for nonlinear dynamics [18, 19]. DMD has recently attracted attention particularly in areas of physics such as fluid mechanics [20] and several engineering fields [24, 36] because of its ability to define a mode that can yield direct information even when applied to time series with nonlinear latent dynamics [20]. The fundamental characteristics of the DMD are considered as the combination of a spatial dimensional reduction method such as POD (in principle, equivalent to principal component analysis) with a Fourier transform in time.

**Fig 1:**
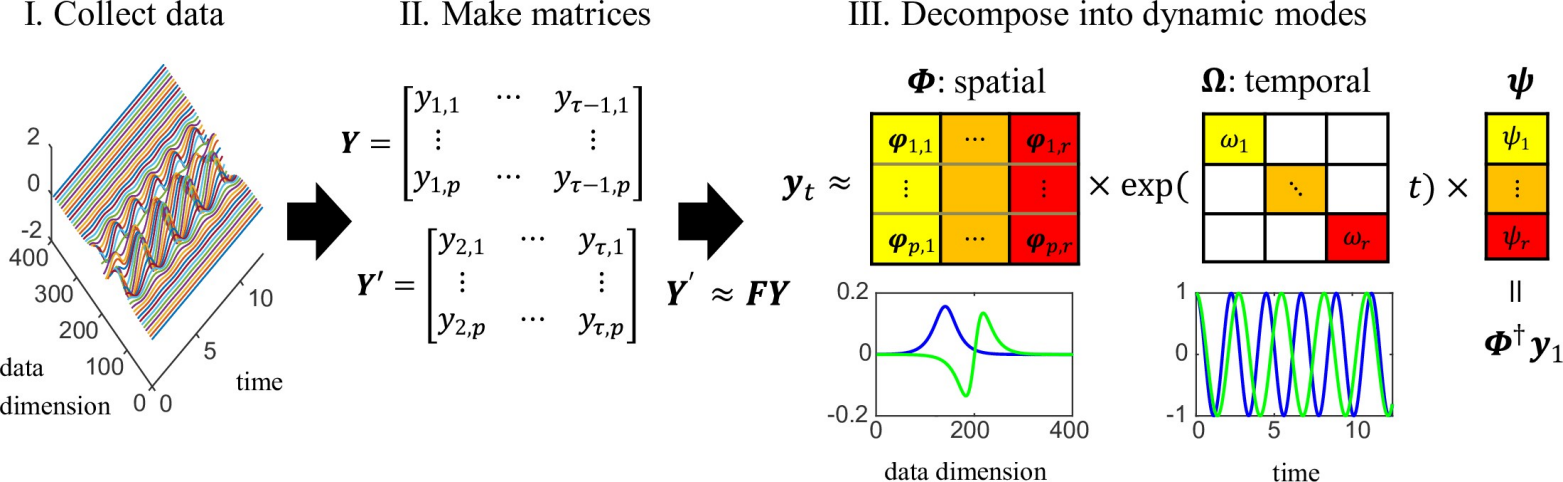
Schematic of the DMD algorithm, applied to a system of nonlinear oscillators. (I) Data are collected from the system, and then (II) two large matrices ***Y*** and ***Y***′ are made. DMD basically assumes ***Y***′ ≈ ***FY*** and (III) performs the eigendecomposition of ***F*** (numerically, $\hat{F}$ below). Then spatial and temporal DMD modes (and coefficients or initial values) are obtained. DMD yields two spatial modes of hyperbolic functions for nonlinear coefficients (blue and green), and two frequency modes of cosine functions (details in S1 Note). Note that these modes can be complex numbers (in this case, the spatial DMD modes are complex), but here we visualized the real part.

To obtain the information about the nonlinear function ***f*** in Eq 1 from data, we consider two sets of data matrix ***Y*** = [***y***_1_,***y***_2_,…,***y***_*τ*−1_] and ***Y***′ = [***y***_2_,***y***_3_,…,***y***_*τ*_], which are composed of observed data sequences $y_{1},y_{2},\ldots ,y_{\tau }({\in C}^{p})$ over time period τ. Note that ***y***_*t*_ = ***g***(***x***_*t*_), where ***g*** is an observation function and ***x***_*t*_ is the latent state. The DMD basically computes the eigendecomposition of the least-squares solution:

$$F≔\underset{\tilde{F}\in R^{p\times p}}{argmin}(1/\tau )\sum_{t=1}^{\tau -1}{\left\|y_{t+1}-\tilde{F}\;y_{t}\right\|}^{2},$$ (2)

i.e., ***Y***′***Y***^†^(= ***F***)(***Y***^†^ is the pseudo-inverse of ***Y***). *p* is the dimension of the data, and *τ* is the time length. The solution to this system may be expressed simply in terms of the eigendecomposition of the matrix ***F***:

$$y_{t}\approx \sum_{j=1}^{p}\varphi_{j}{\exp\left(\omega_{j}t\right)\psi }_{j}=\Phi \exp\left(\Omega t\right)\psi ,$$ (3)

where ***φ***_*j*_ and ***Φ*** are the DMD mode vector and matrix defined by using an eigenvector of ***F***, respectively. *ψ*_*j*_ and ***ψ*** are a scalar and vector coefficient of the DMD mode, respectively. **Ω** = diag(*ω*_*j*_) is a diagonal matrix whose entries *ω*_*j*_ are calculated by ln(*λ*_*j*_)/Δ*t*, in which *λ*_*j*_ is an eigenvalue of ***F*** and Δ*t* is a time interval of the discrete-time system. These are illustrated in Fig 1 with an example of a nonlinear oscillator system (details in S1 Note). Note that *ω*_*j*_ indicates an angular frequency but the temporal frequency (commonly referred to as frequency) using the analysis below is computed by *f*_*j*_ = ln(*λ*_*j*_)/2πΔ*t*.

To solve the problem in Eq 2 to obtain Eq 3, practically, the matrix ***F*** may be intractable to analyze directly when the state dimension is large [22]. In this case a rank-reduced representation in terms of a POD-projected matrix $\hat{F}$ has been used for the basic DMD algorithm [37, 38]. First, the singular value decomposition (SVD) of ***Y*** is performed:

$$Y\approx U\Sigma V^{*}$$ (4)

where * denotes the conjugate transpose, $U\in C^{n\times r}$ (POD modes), $\Sigma \in C^{r\times r},\;V\in C^{\tau \times r}$, *r* is the rank of the reduced SVD approximation. The matrix ***F*** may be obtained by using the pseudoinverse of ***X*** obtained via SVD:

$$F=Y^{\prime }V\Sigma^{-1}U^{*}.$$ (5)

To compute $\hat{F}$ efficiently, the projection of the full matrix ***F*** onto POD modes can be obtained:

$$\hat{F}=U^{*}FU=U^{*}Y^{\prime }V\Sigma^{-1}.$$ (6)

Then, we compute the eigendecomposition of $\hat{F}$ and obtain the above eigenvalue **Λ** = diag(*λ*_*j*_) and a matrix of eigenvector ***W***. The DMD modes are given by the columns of ***Φ***:

$$\Phi =Y^{\prime }V\Sigma^{-1}W\Lambda^{-1}.$$ (7)

For the remaining vector coefficient of the DMD mode ***ψ***, when considering the initial snapshot ***y***_1_ at the time *t*_1_, Eq 3 gives ***y***_1_ = ***Φψ***. Then, the vector coefficient can be calculated as ***ψ*** = ***Φ***^†^***y***_1_. For this reason, the DMD modes and their coefficients have a spatial property related to a given temporal frequency (Fig 1 right bottom) with a growth or decay rate. Note that in this framework, we do not need the prior knowledge about the dynamical property of the input time-series matrix, but we need to the knowledge about the input time-series data. For example, we used inter-agent distances in this study because we have already know that the distances are critical for the multi-agent systems, but in general, this would be depends on the problem.

#### Dynamic mode decomposition with reproducing kernels

When the collective motion has time-varying interaction modes like systems of actual organisms, the original DMD may be difficult to apply. Generally, the original DMD has numerical disadvantages, related to the accuracy of the approximate expressions derived from the data. A number of variants have been proposed to address this shortcoming [37, 38]. These decomposition methods have been generalized to a reproducing kernel Hilbert space (RKHS) [30], called *DMD with reproducing kernels*. This modified decomposition method can implicitly approximate the dynamics with (theoretically) infinite basis functions even with low-dimension sequences and transiently changing behaviors.

In DMD with reproducing kernels, let H be the RKHS embedded with the dot product determined by a positive definite kernel *k*. Then, we consider the Koopman operator $K_{H}$: H→H [31], which is an infinite-dimensional linear operator acting on the feature map ϕ:M→H. That is, it maps *ϕ* to the new function $K_{H}\phi$ as follows:

$$K_{H}\phi =\phi \circ f,$$ (8)

where ∘ denotes the composition of *ϕ* with ***f***. We denote *ϕ*_***x***_ as an instance of *ϕ* with respect to ***x***. First, as in the case of the original DMD, we robustify the DMD with reproducing kernel by POD projection (i.e., perform kernel PCA). Here we consider the matrices $M_{1}≔[\phi_{x_{1}},..,\phi_{x_{\tau -1}}]$ and $M_{2}≔=[\phi_{x_{2}},..,\phi_{x_{\tau }}]$. The Gram matrix *G*_*yy*_ of the kernel *k*(***y***_*i*_,***y***_*j*_) is defined at ***y***_*i*_ and ***y***_*j*_ (*i* and *j* are the time points) of the observation data matrix ***Y***. Similarly, the Gram matrix *G*_*yy*′_ of the kernel between ***Y*** and ***Y***′ can be calculated. At this time, the Gram matrix $G_{yy}=M_{1}^{*}M_{1}$, where $M_{1}^{*}$ indicates the Hermitian transpose of $M_{1}$ (and also $G_{yy\prime }=M_{1}^{*}M_{2}$). Then, a centered Gram matrix is defined by $\bar{G}=HGH$, where *G* is a Gram matrix, **H** = **I** − **1***τ*, **I** is a unit matrix, and **1***τ* is a *τ*-by-*τ* matrix, for which each element takes the value 1/*τ*.

Here, suppose that the eigenvalues and eigenvectors can be truncated by $\bar{G}\approx \bar{B}\bar{S}{\bar{B}}^{*}$ where *p* (≤*τ*) eigenvalues are adopted. Then, a matrix of the principal orthogonal direction in the feature space arranged in a row is given by $U=M_{1}H\bar{B}{\bar{S}}^{-1/2}$. Since $M_{2}=K_{H}M_{1}$, the projection of $K_{H}$ onto the space spanned by the principal orthogonal direction is given as follows:

$$\hat{F}=U^{*}K_{H}U={\bar{S}}^{-1/2}{\bar{B}}^{*}H(M_{1}^{*}M_{2})H\bar{B}{\bar{S}}^{-1/2}.$$ (9)

Note that ${M_{1}^{*}M}_{2}=G_{yy\prime }$ is computable. Then, if we let $\hat{F}={\hat{T}}^{-1}\hat{\Lambda }\hat{T}$ be the eigendecomposition of $\hat{F}$, we obtain the centered DMD mode ${\bar{\varphi }}_{j}=Ub_{j}=M_{1}H\bar{B}{\bar{S}}^{-1/2}b_{j}$, where ***b***_*j*_ is the *j*th row of ${\hat{T}}^{-1}$. The diagonal matrix $\hat{\Lambda }$ comprising the eigenvalues represents the temporal evolution of the mode.

A direct and important application of this analysis in the feature space is the embedding and classification of dynamics using extracted features. The set of Koopman spectra estimated from the analysis can be used as the basis for a low-dimensional subspace that represents the dynamics. Generally, selection of an appropriate representation of the data to reflect the structure is a fundamental issue in pattern recognition. Among several kernels to reflect time-series data structure, we previously proposed the Koopman spectral kernels [32] (see Materials and Methods). For example, our previous work succeeded in the classification into the type of human locomotion from motion capture data [30, 32], using feature vectors determined by the Koopman spectral kernels. Although the above algorithm cannot extract a feature directly from the data space because of its decomposition in feature space, we used some techniques to estimate the difference between the original data and the reconstructed sequence in data space (see S3 Note).

## Results

### Application to schooling behavior in explicit model

**Fig 2:**
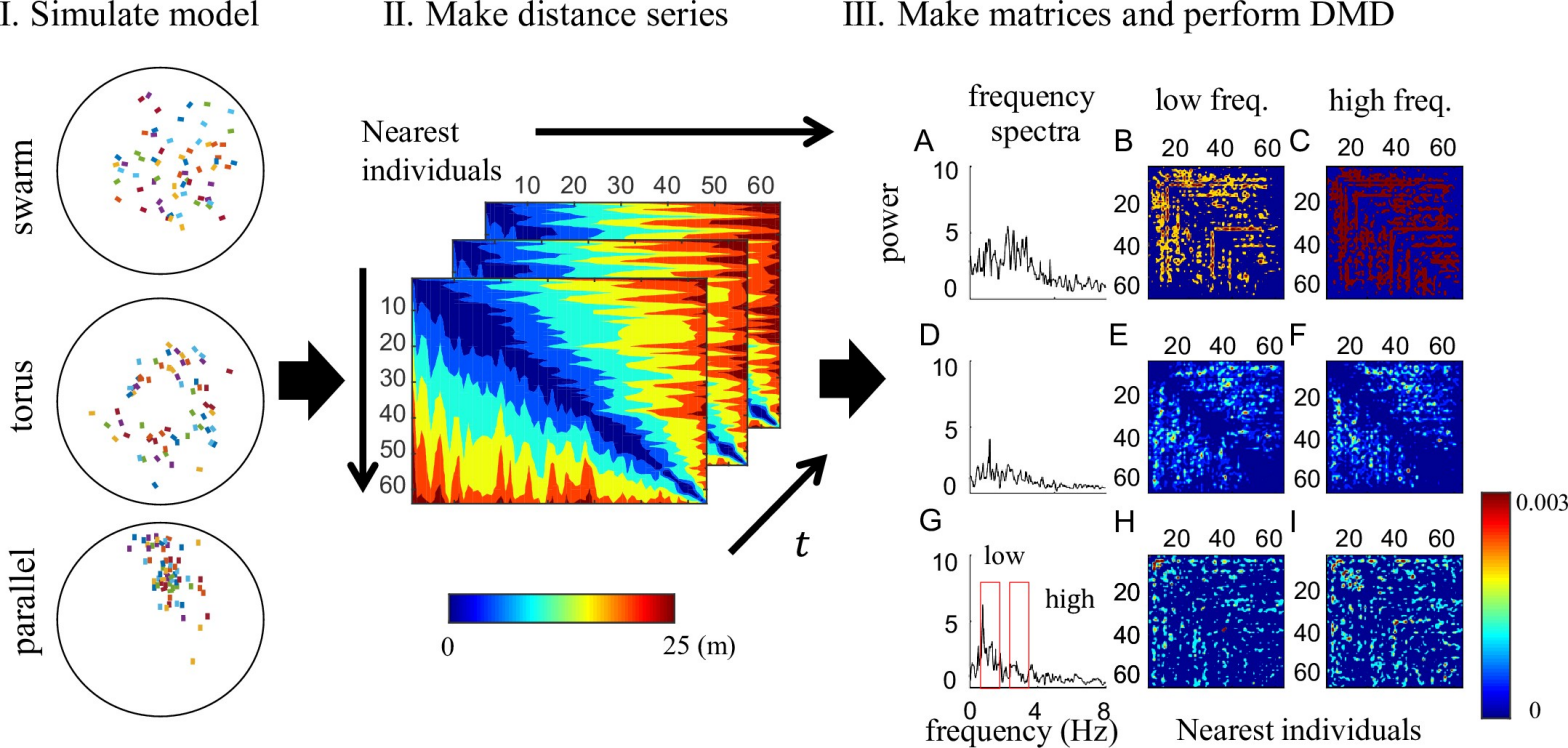
Dynamic modes of simulated fish-schooling data. To apply DMD to collective motions, we used a simple schooling simulation model (I) to create three different behaviors from changes in only one parameter (for details, see Materials and Methods). Then we computed the distance series with respect to nearest individuals to represent the topological relationships among individuals (II). We transformed this series into matrix form to squeeze the distance matrix. After DMD, we obtain temporal and corresponding spatial modes (III). The temporal DMD modes (A, D, G) are shown in the spectra as a function of temporal frequency. Using the results of the frequency spectra (A, D, G), we separated the spatial modes into low (0.5–1.5 Hz) and high (2–3 Hz) frequency domains. As the temporal frequency spectra, the power spectra of the spatial DMD modes for the swarm (B-C), parallel (H-I), and torus behavior (2E-F) were stronger in this order.

First, we applied the basic DMD to a simple model of schooling (see Materials and Methods and Fig 1) to verify that our approach can extract and discriminate the global dynamics of collective motions from observed data without the prior knowledge about the labeled motions. Although the model only employs the simple and explicit local interaction rules of repulsion, alignment, and attraction [2], complex collective motion emerges because of the resultant many-body interactions. For example, changes in the parameter interaction rule *r*_*o*_, which is the radius of the zone of orientation between the repulsion and attraction zones (detail in Materials and Methods), can determine whether the collective motion takes the form of a swarm, torus, or parallel behavior (Fig 2 left and S1–S3 videos: *r*_*o*_ increasing in this order). For generating the three distinct behavioral shapes, we adopted the simple model [2] only based on the specific distance called *metric* framework, rather than more realistic model based on the specific number of agents called *topological* framework [39, 40] or the mixture model both with the metric and topological framework [41]. Note that the particles moved at a constant speed (4 m/s with an individual variance). This indicates that the particles are not being driven by Brownian motion. DMD can extract spatially coherent (global) dynamic modes and also can estimate dynamic properties of the local interactions among the agents in a group. We show the results using a distance-matrix time series between individuals and sorted by nearest neighbors (Fig 2 middle) at each time indicating three distinct dynamical properties (the results inputting the distance with fixed individuals and the raw Cartesian coordinates in S4 Fig do not show the distinct properties). It should be noted that input matrix sorted by nearest neighbors can more stably compute the DMD than the unsorted matrix (see S4 Fig), because the temporal change in the distance matrices are more stable in the sorted matrix than the unsorted one (see S1 Fig). In the sorted matrix, we consider that the simulated agents do not discriminate each other (the orders of the component of the matrix change over time) as well as the metric (and topological) framework. Moreover, noted that also in the metric framework, the neighbor agents move similarly in the specific zone (i.e., zone of orientation) as well as in the topological framework. In case of the nearest sorted distance matrix, the results in temporal DMD mode exhibited a relatively wide and strong spectrum, a wide and weak spectrum, and a narrow but strong spectrum for the swarm, torus, and parallel behaviors, respectively (Fig 2A, 2D and 2G). For the spectrum of the parallel behavior, low frequency (0.5–1.5 Hz) peaks may indicate alignments and transient interactions resulting from collision with the wall, because the additional simulations without a boundary condition caused the high-frequency peak to vanish (S3 Fig).

The power spectra of the spatial DMD modes [24] averaged by the DMD modes within the above frequency interval also show the distinct spatial properties for the three different behavioral shapes in the low (0.5–1.5 Hz) and high frequency (2–3 Hz) intervals. As the temporal frequency spectra, the power spectra of the spatial DMD modes for the swarm (Fig 2B and 2C), parallel (Fig 2H and 2I), and torus behavior (Fig 2E and 2F) were stronger in this order. Especially, the power spectra of the spatial DMD modes of the torus behavior were weak near the diagonal elements, i.e., nearest agents to each other. Note that all the DMD reconstructions were performed sufficiently (see S2 Note and S5 Fig).

**Fig 3:**
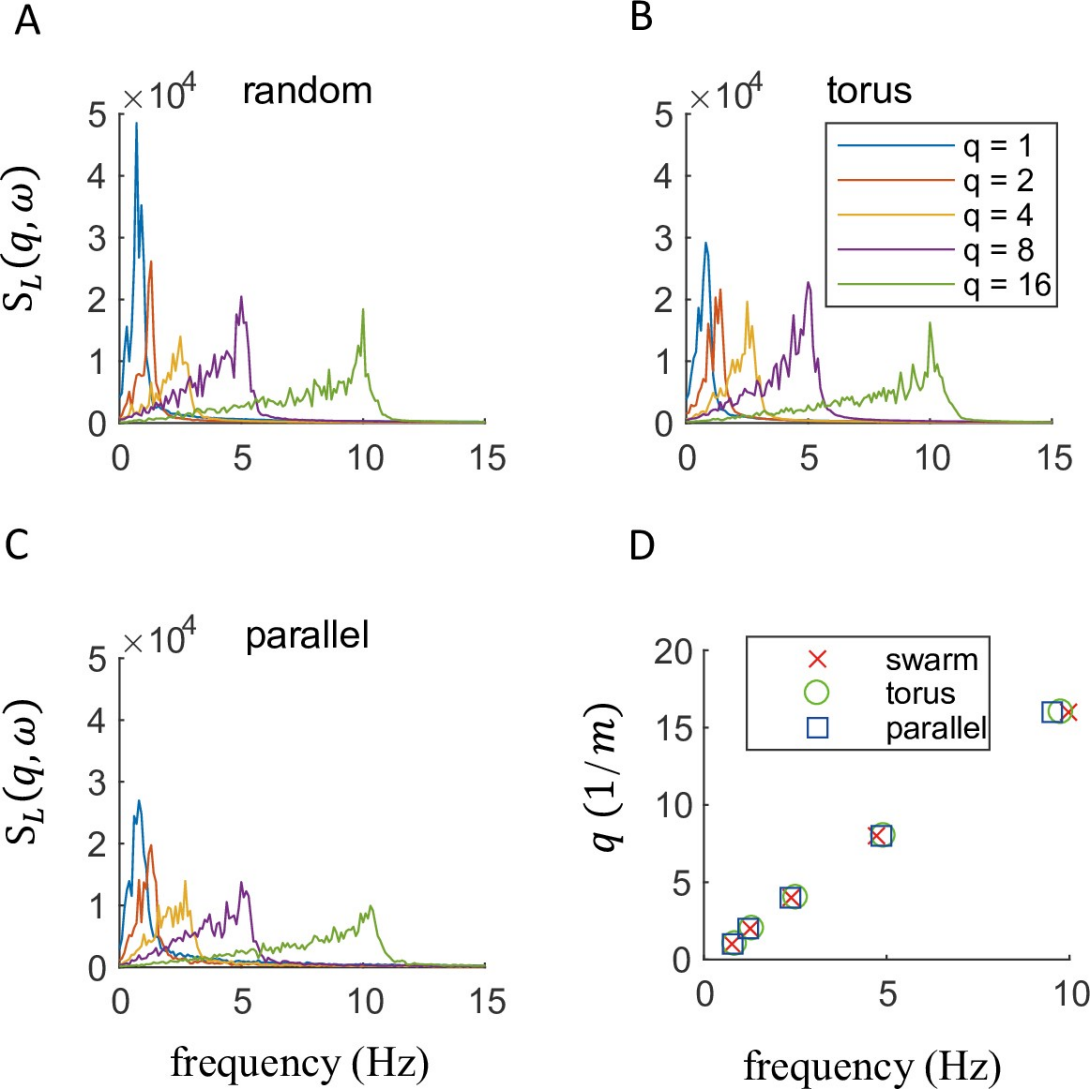
Longitudinal dispersion relations in schooling motion. (A-C) The longitudinal dynamic structure factor *S*_*L*_(*q*,*ω*) in the swarm, torus, and parallel behavior are shown, where *q* is the wavenumber and *ω* is the temporal frequency. These had similarly distinct peaks for each wavenumber, and (D) the dispersion relations between wavenumber and frequency in those peaks were almost linear and equal among all collective motions. Results for the transverse dynamic structure factor are provided in S4 Fig.

From a more general perspective, we examined wave properties of wavenumber and frequency using the longitudinal and transverse dynamic structure factors [26, 29] which is a Fourier transform of the density-density correlation function in both space and time, and can separate into longitudinal and transverse modes [27] (see Materials and Methods). This analysis revealed that the longitudinal dynamic structure factor had shifting peaks for each wavenumber under the three types of behavior (Fig 3A–3C), and the dispersion relations between wavenumber and frequency in spectrum peaks were almost linear and equal among all collective motions (Fig 3D). This finding indicates that the three motions had pseudo-acoustic mode dynamics or sound-like propagation modes [42]. Similarly, for the transverse dynamic structure factor, the dispersion relations were nearly linear (S6 Fig), suggesting that the collective motion also shared properties with transverse waves, which did not occur (or was damped) in a normal fluid. Thus, the collective motions in this study are similar to the motion of a viscoelastic fluid. These dispersion relations were independent of the type of the emergent motion (even without a boundary, as in S7 Fig) and suggest that the wave properties in multiple spatiotemporal scales cannot discriminate the type of motion that emerges. Instead, DMD analysis can extract the properties of dynamic interaction for the different collective motions. Additional discrimination results including the existing specific parameters such as polarization and angular momentum [2] are shown in S10 Fig and S2 Note. It should be noted that for the discrimination, the advantage of our method is that we do not need the prior knowledge about the labeled group behaviors.

### Application to actual strategic collective motion

**Fig 4:**
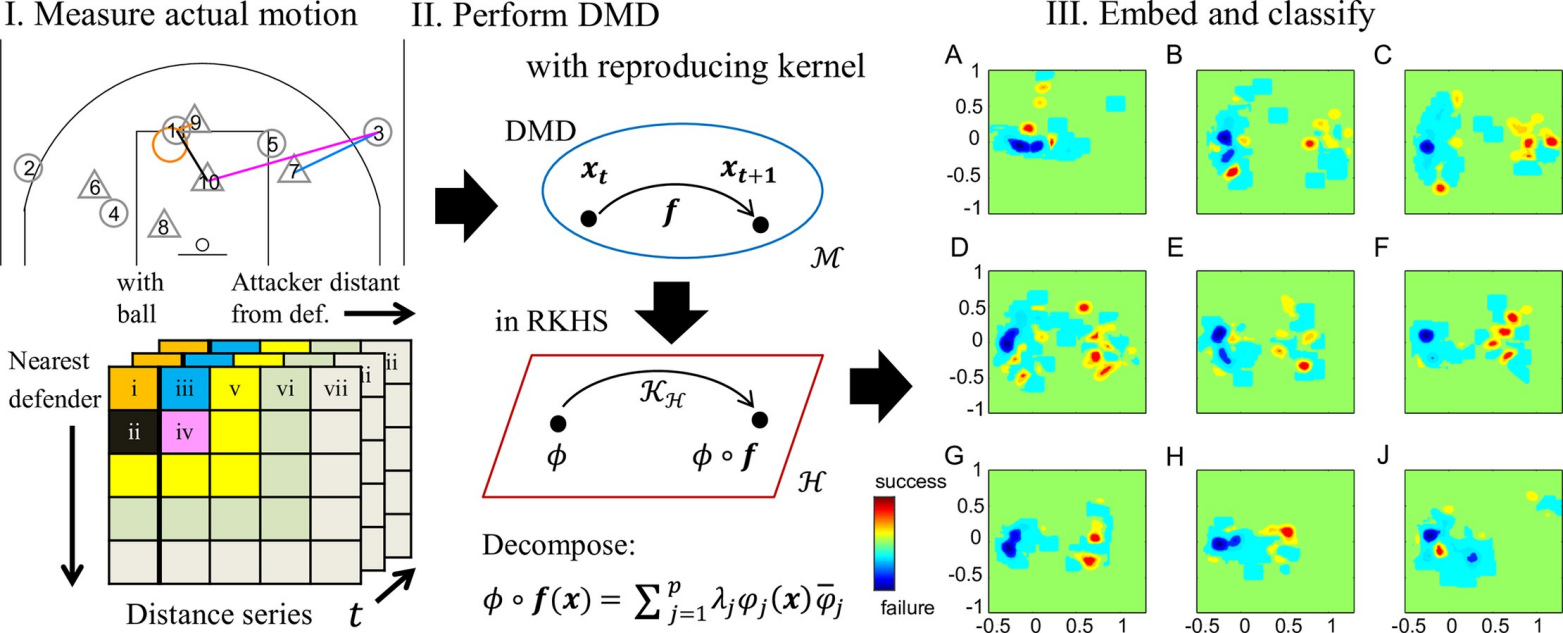
DMD with reproducing kernel applied to multiagent sports. (I) Data is measured from an actual sporting behavior, and then the attacker-defender distance matrices are computed. The leftmost column lists the most important distances between the attacker nearest to the ball and defenders near that attacker starting with the nearest neighbor at the top. The remaining columns are sorted in the order of attackers with the most separation from their defenders. We assume that the separation between attackers and their corresponding defenders is important for an attacker’s chance of scoring, so these separations are input distances for the DMD with reproducing kernels (II). The method expands DMD from the data space into a feature space called reproducing kernel Hilbert space (RKHS). In the feature space, the Koopman operator $K_{H}$ is decomposed into the Koopman eigenvalue *λ*_*j*_, Koopman eigenfunction *φ*_*j*_(***x***), and the Koopman mode ${\bar{\varphi }}_{j}$. Using the decomposed properties, we computed Koopman spectral kernels to express the similarities between the attack segments. (III) shows embedding via visualization methods called multidimensional scales with the distance matrix of the Koopman spectral kernels (see Materials and Methods) contoured by frequencies of success (red) and failure (blue) of the shot. (A-G) correspond to the results inputting the distances in (i-vii) of (I). (H) and (J) show the results inputting the Euclidean distance and Cartesian coordinates, respectively. The best case of inputting four relevant attacker-defender distances (D) shows high expressiveness for scoring probability due to its wide distribution across the plot.

As an application to actual complex collective behavior in a low-dimension input space, we used player-tracking data from actual basketball games. Using DMD with reproducing kernels, we extracted the dynamic interaction properties, and predicted the probability of a shot (Fig 4). Position data was comprised of the horizontal Cartesian positions of every player and the ball on the court, recorded at 25 frames per second. We defined an attack segment as the period beginning when all players enter the attacking side of the court and ending before a shot was made (77 shots were successful out of 192 attack segments). We focused on the most effective attacker-defender distances (previously shown in [9]), which were temporally and spatially corrected to predict the success or failure of the shot (Fig 4 left). Although all of the distances were expressed in 25 dimensions (five attackers and defenders), we previously reduced such data to four dimensions [9]: ball-mark distance (i.e., the maximum distance between the attackers with the ball and the nearest defenders), ball-help distance (i.e. the secondary maximum distance of the ball-mark distance), pass-mark distance (i.e., the maximum distance between the attackers without the ball and the nearest defenders), and pass-help distance (i.e. the secondary maximum distance of the pass-mark distance) (Fig 4 left).

To apply DMD with reproducing kernels and verify its predictive performance, we used nine input matrices: (i-iv) one- to four-dimensional critical distances as described above. For additional verification, (v-vii) 9, 16, 25 distances in Fig 4 left and (viii) 25-dimensional Euclidean distance matrices without spatiotemporal corrections were calculated. We also used (ix) the Cartesian positions (total 20 dimensions) of all ten players (typical time series are shown in S8 Fig). Note that reconstructions from the DMD with reproducing kernel outperformed those from the original DMD (S3 Note and S9 Fig).

**Fig 5:**
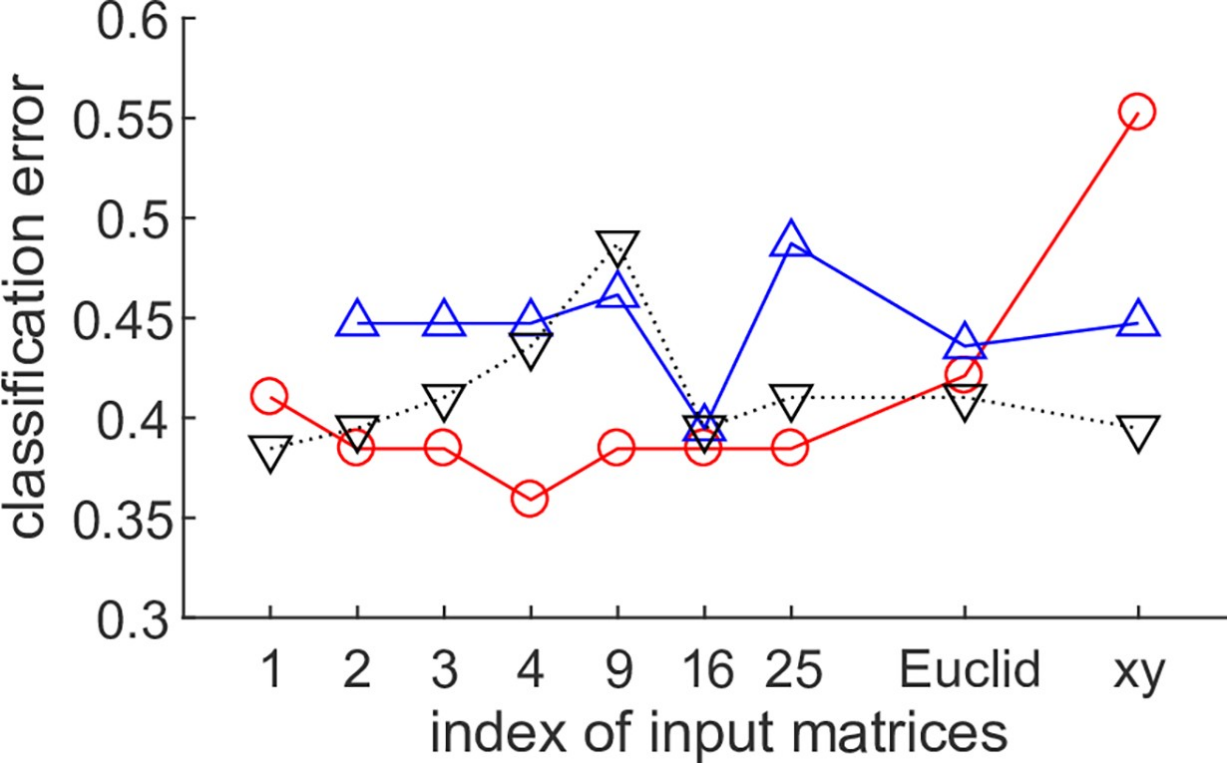
Prediction performance of DMD with reproducing kernels. This plot shows the error of naive Bayes classifier for success or failure of the shot, using the DMD with reproducing kernels (red), the DMD (blue), and the maximum values (black) inputting various input matrices. 1 to 25 indicates the number of input distance series shown in Fig 4 left (i to vii). The Koopman spectral kernel derived by inputting four relevant inter-player distances achieved the minimum classification error of 35.9%. Overall, the Koopman spectral kernels produced better classifications than the kernels of the original DMD and the maximum values. The kernel of the original DMD using only one distance was not computed because of its low expressiveness.

For predicting the outcome of a team’s attacking movement (i.e., shot), we computed the probability of the shot outcome because the shot outcome is probabilistic rather than deterministic. Thus, we used classifiers outputting the posterior probability such as a naive Bayes classifier (results with other classifiers are described in S3 Note and S11 Fig). The feature vectors inputting the classifier were created by Koopman spectral kernels [32] (See Materials and Methods) to express the similarities between the attack segments with various input distances. For comparison, the kernel of the basic DMD and the simple feature vector using the maximum adjusted distances in the previous study [9] were also computed. Fig 5 shows the results of applying a naive Bayes classifier. The horizontal axis represents the nine input matrices and the vertical axis the classification error (this is the median value of 5-fold cross-validation error). Overall, the Koopman spectral kernels produced better classifications than the kernels of the DMD and the feature vector using the maximum distance (and Cartesian coordinates). If the decomposition methods cannot extract the dynamics (i.e., the result had a low expressiveness), the classification error is near the chance level (0.5) such as when DMD using 25 distances and DMD with reproducing kernels using the Cartesian coordinates. Especially, the Koopman kernel principal angles derived by inputting four important distances exhibited the minimum classification error of 35.9%. Additionally, we examined the error analysis of all 192 attack-segments of the best classifier: the numbers of true positives, true negatives, false positives, and false negatives were 22, 97, 18, and 55, respectively (Typical examples are shown in S5–S8 Videos). Note that the classification error was computed as the median values of the five test sets and then did not correspond to the computation from the above values. Our best classifier tended to predict non-score (152: true and false negatives) more than the actual (115).

Fig 4 right shows embedding via multidimensional scale with the distance matrix of the Koopman spectral kernels, contoured by frequencies of success and failure of the shot. Figs (III) A-G correspond to the results inputting the distances in (i-vii) of Fig 4(I). Fig 4H and 4J show the results inputting the Euclidean distance and Cartesian coordinates, respectively. For example, the best case of the four important attacker-defender distances (Fig 4D) showed high expressiveness for scoring probability due to the plot’s wide distribution. In contrast, the plots were less widely distributed when only a single distance (Fig 4A) or the Cartesian coordinates of all players (Fig 4J) were used. The kernels of the basic DMD showed even less distribution and less expressiveness (S12 Fig).

## Discussion

Our objectives in this study were to verify the application of DMD to simple collective motion models and develop the method for more complex and low-dimensional actual motions. First, the DMD applied to a simulation of an explicit model of collective motion extracted different temporal frequency modes with spatial interaction coherence among three emergent motions (Fig 2). These wave properties at multiple spatiotemporal scales showed similar dispersion relations (Fig 3). The three schooling behavior differed only by one parameter, the radius of the orientation zone, which explains why the wave properties were invariant. Analysis of the dynamic structure factor revealed that schooling motions have viscoelastic properties [27], and this quality was independent of the type of the emergent motion. However, the visual appearances of the three emergent motions are distinct, and these differences are generated by dynamic changes in local interactions in response to changing circumstances, including other agents. DMD extracted the dynamic properties to explain the difference among the three global behaviors. In general, DMD can extract interactive coherence spectra with various temporal frequency modes for an active matter or non-equilibrium many-body system as long as interaction rules are consistent. However, note that transient behaviors such as occurs with actual organism motion in a small group are difficult to reconstruct with the original DMD method [22].

We also developed DMD with reproducing kernels [30, 32] for applications to small group behavior with transient behavioral rules. We mapped the latent dynamic characteristics to the feature space with high expressiveness, and obtained intuitively reasonable outcomes by successfully predicting the achievement of the group objective. Compared with the original DMD, the advantages of DMD with reproducing kernels were that it can be applied to low-dimensional data and transient behaviors. Competitive collective motion among small groups that can dynamically change their strategy according to the situation is just such a situation. Specifically for a dataset from basketball games, the highest prediction performance was achieved using a limited set of relevant player distances (Figs 4 and 5). The scoring outcome can be predicted by discarding extra information so that the remaining data is more semantically important. The vector series reflects four characteristics: the scoring probability of a player in the (i) shot, (ii) dribble to goal, and (iii) pass, and (iv) the scoring probability of a dribbler after the pass. The proposed kernels reflected the time series of all interactions between players and were more effective for classification than the kernels based on the information only about the shot itself or the raw Cartesian positions. Well-trained ballgame teams aim to create scoring opportunities by continuously selecting strategic passes and dribbles or by improvising when no shooting opportunity is available.

However, our proposed method has some drawbacks. In original DMD for the fish-schooling model, we could not extract the dynamic interaction modes for centripetal motion in the torus behavior. Theoretically, when motion deviates greatly from the ideal oscillator, the mode may not be extracted by DMD. Also in DMD with reproducing kernels applied to the basketball data, even the best classification was not very accurate, with only 64.1% accuracy. Our framework may have neglected two factors. The first is the existence of local interactions between players, such as local competitive and cooperative play by the attackers and defenders [9, 43] which should be examined in higher spatial resolution than was available in our data. A better model would reflect the hierarchical dependencies of global and local dynamics. The second is the limitation of the input matrices of two-agent distances. To achieve more accurate classifiers, hand-made input vector series such as Cartesian coordinates or specific movement parameters should be used in addition to the most important input factor of inter-player distances.

Overall, we developed a method to predict and classify collective motion dynamics without equations by decomposing data into several temporal frequency modes with coherence among spatial interactions. This algorithm can, in general, be applied to the analysis of the complex dynamics in groups of living organisms or artificial agents, which currently eludes formulation. This method can provide us to predict the outcome of unknown behavior from collective movement data in non-trivial dominance law such as active matters. From a different viewpoint, the rule-based fish-schooling model and human group in a sport used in this study can be considered as the examples in which the communication is likely to be measured in a physical space. Therefore, if we can measure the outputs of the communication and suppose that there are underlying dynamics behind the obtained data, our approach can deal with various means of communication between the agents. Practically, in various material and life sciences or human community, supervisors (experimenters, coaches or teachers) spend considerable amounts of time analyzing the collective motion in the domain. Application of a system, such as the one presented here, may create useful plans that are currently derived only from their implicit experience.

## Materials and methods

### The configuration of the schooling model

The schooling model we used in this study was a unit-vector based (rule-based) model, which accounts for the relative positions and direction vectors of other fish agents, such that each fish tends to align its own direction vector with those of the members. We used an existent model [44] based on the previous work [2]. The specific parameters are shown in S1 Table. In this model, *N* = 64 agents are described by a two-dimensional vector with a constant velocity (4 m/s) in a boundary circle (radius: 25 m) as follows:

ri=(xiyi), (10)

$$v_{i}(t)=|v_{i}|d_{i},$$ (11)

where ***d***_i_ is an unit vector. At each time step, a member will change direction according to the positions of λ neighbors. In this study, to generate the three distinct behaviors (the swarm, torus, and parallel behaviors), we simply used the metric framework, i.e., the agents change direction according to the positions of the neighbors. The space around an individual is divided into three zones with each modifying the unit vector of the velocity

The first region, called the repulsion zone with a radius *r*_*r*_, corresponds to the “personal” space of the particle. Individuals within each other’s repulsion zones will try to avoid each other by swimming in opposite directions. The second region is called the orientation zone, in which members try to move in the same direction (radius *r*_*o*_). We changed the parameter *r*_*o*_ to generate the three behavioral shapes. Next is the attractive zone (radius *r*_*a*_), in which agents swim towards each other and tend to cluster, while any agents beyond that radius have no influence.

Let λ_r_, λ_o_, and λ_a_ be the numbers in the zones of repulsion, orientation and attraction respectively. For λ_r_ ≠ 0, the unit vector of an individual at each time step τ is given by:

$$d_{i}\left(t+\tau ,\lambda_{r}\neq 0\right)=-\left(\overset{\lambda_{r}}{\underset{j=1}{\sum }}\frac{r_{ij}\left(t\right)}{\left|r_{ij}\left(t\right)\right|}\right),$$ (12)

where |***r***_*ij*_| = |***r***_*j*_ − ***r***_*i*_|. The direction vector points away from neighbors within this zone to prevent collisions. This zone is given the highest priority; if and only if λ_r_ = 0, the remaining zones are considered. The unit vector in this case is given by:

$${\vec{d}}_{i}\left(t+\tau ,\lambda_{r}=0\right)=-\frac{1}{2}\left(\overset{\lambda_{o}}{\underset{j\neq i}{\sum }}{\vec{d}}_{i}\left(t\right)+\overset{\lambda_{a}}{\underset{j\neq 1}{\sum }}\frac{{\vec{r}}_{ij}\left(t\right)}{\left|{\vec{r}}_{ij}\left(t\right)\right|}\right),$$ (13)

The first term corresponds to the orientation zone while the second term corresponds to the attraction zone. The above equation contains a factor of 1/2 which normalizes the unit vector in the case that both zones have non-zero neighbors. If no agents are found near any zone, then the individual maintains constant velocity at each time step.

In addition to the above, we constrain the angle by which a member can change its unit vector at each time step to a maximum of *β*. This condition was imposed to facilitate rigid body dynamics. Because we assumed point-like members, all information about the physical dimensions of the actual fish is lost, which leaves the unit vector free to rotate at any angle. In reality, however, conservation of angular momentum will limit the ability of the fish to turn angle *θ* as follows:

$$\begin{array}{c} \mathrm{If}\theta >\beta ,\;d_{i}\left(t+\tau \right)\cdot d_{i}\left(t\right)=\cos\left(\beta \right)\\ Otherwise\;d_{i}\left(t+\tau \right)\cdot d_{i}\left(t\right)=\cos\left(\theta \right). \end{array}$$ (14)

If the above condition is not met, the angle of the desired direction at the next time step is rescaled to *θ* = *β*. In this way, any un-physical behavior such as having a 180° rotation of the velocity vector in a single time step, is prevented.

### Simulation of the model

Initial conditions were set so that the particles would generate the torus motion, though all three motions emerge from the same initial conditions. The initial positions of the particles were arranged using a uniformly random number on a circle with a uniformly random radius between 6 m and 16 m (the original point is the center of the circle). The initial velocity was set to be perpendicular to the initial position vector. We modeled schooling behavior with and without circular boundary conditions (the main results in Fig 2 used a circular boundary of 25 m radius). The average values of the control parameter *r*_*o*_ were set to 2, 10, and 13 to generate the swarm, torus, and parallel behaviors, respectively. Although the previous study of the model [2] examined the effect of the noise added to various parameters, we simply added noise to the constant velocities among the agents (but constant within a particle) with a standard deviation of σ to generate the three distinct behavioral shapes with a certain variability. If the noise is close to zero, the group behavior has less variability and if the noise increases, the group behavior might fragment; thus we set σ to 0.05 according to the previous settings [44]. The time step in the simulation was set to 10^−2^ s. We simulated 15 trials for each parameter *r*_*o*_ in 10 s intervals (1000 frames). The analysis start times were varied depending on the behavior type to avoid calculating the transition period (torus: 10 s, swarm and parallel: 30 s after the simulation start). We customized freely available MATLAB code for the simulation [44].

### Dynamic structure factor

We calculated the longitudinal and transverse dynamical structure factor [27], *S*_*L*_(***q***,*ω*) and *S*_*T*_(***q***,*ω*), respectively, given as

$$S_{\alpha }\left(q,\omega \right)=\frac{1}{2\pi N}\int dt\langle j_{\alpha }\left(q,t\right)\cdot j_{\alpha }\left(-q,0\right)\rangle \exp\left(i\omega t\right),$$ (15)

where *α* is *L* and *T*, and ***q*** is the wave vector, *ω* is the angular frequency, and ***j***_*α*_ is the density-density correlation function in both space and time [26, 29]:

$$\begin{array}{c} j_{L}\left(q,t\right)=\sum_{i}\left(v_{i}\left(t\right)\cdot \hat{q}\right)\hat{q}\exp(iq\cdot r_{i}\left(t\right)),\\ j_{T}\left(q,t\right)=\sum_{i}\left(v_{i}\left(t\right)-\left(v_{i}\left(t\right)\cdot \hat{q}\right)\cdot \hat{q}\right)\exp(iq\cdot r_{i}\left(t\right)), \end{array}$$ (16)

where $\hat{q}=q/|q|$ and ***r***_*i*_ is the two-dimensional position vector.

Results revealed that the longitudinal dynamic structure factor had a shifted peak among the wavenumbers (Fig 3A–3C). These peak positions *ω* shift followed a Brillouin-like dispersion relation:

$$\omega_{shift}=c\left|q\right|,$$ (17)

where *c* is a coefficient which has the dimension of velocity, and represents the soundwave velocity in conventional applications of Brillouin peaks. We can, therefore, say that these systems possess pseudo-acoustic mode dynamics or a soundlike propagation mode for agent density. The sound velocity of this pseudo-acoustic mode can be calculated from the dispersion relation, shown in the Fig 3D.

Results revealed that the longitudinal dynamic structure factor had shifted peaks among wavenumbers (Fig 3A–3C), and the dispersion relations between wavenumber and frequency at those spectral peaks were almost linear and equal among all collective motion types (Fig 3D). Similarly, for the transverse dynamic structure factor, the dispersion relations were also almost linear, although it was not relatively clearer than the longitudinal one (S6 Fig). These dispersion relations were independent of the type of the emergent motions (even without the boundary: S7 Fig).

### Group sport data

We used player-tracking data from two actual international basketball games in 2015 collected by the STATS SportVU system. The total playing time was 80 min, and the total score of the two teams was 276. Positional data comprised the Cartesian position of every player and the ball on the court, recorded at 25 frames per second. We eliminated transitions to attacking to automatically extract the time periods to be analyzed (called an *attack-segment*). We defined an attack-segment as the period starting when all attacking players enter the active half of the court and ending 1 s before a shot on goal was attempted. We analyzed a total of 192 attack-segments, 77 of which ended in a successful shot.

We focused on the effective attacker-defender distances (previously shown by [9]), which were temporally and spatially corrected to predict the success or failure of the shot. Although all of the distances comprise 25 dimensions of data, we reduced the dimensions to four dimensions in previous work [9]: ball-mark distance, ball-help distance, pass-mark distance (i.e., the maximal distance between the attacker without the ball and the nearest defenders), and pass-help distance (i.e., the second maximal distance of pass-mark distance) (Fig 4 left).

For applying DMD with reproducing kernels and its verification, we used nine input matrices: (i-iv) one to four-dimensional critical distance of the above, and for verification, (v) total distance and (vi) 25-dimensional Euclidean distances without spatiotemporal correction were calculated. We also used (vii) the Cartesian coordinates (total 20 dimensions) of all the ten players (typical time series are shown in S8 Fig).

### Reproducing kernel of the DMD

In performing the DMD with reproducing kernel, we used the Gaussian kernel or radial basis function:

$$k\left(y_{i},y_{j}\right)=exp\left(-\frac{\|y_{i}-y_{j}\|{}^{2}}{2\sigma \prime^{2}}\right),$$ (18)

where *i* and *j* are the time point of the observation data, and *σ*′^2^ is the kernel width set as the median of the distances from a data matrix [30, 32]. For example, the Gram matrix *G*_*yy*_ in the main text of this Gaussian kernel can be defined as follows:

$$G_{yy}=\left(\begin{array}{ccc} k\left(y_{1},y_{1}\right) & \ldots & k\left(y_{\tau -1},y_{1}\right)\\ \vdots & \ddots & \vdots \\ k\left(y_{1},y_{\tau -1}\right) & \ldots & k\left(y_{\tau -1},y_{\tau -1}\right) \end{array}\right).$$ (19)

### Koopman spectral kernels

Selection of an appropriate representation of the data is a fundamental issue in pattern recognition. The important point is to design features (i.e., kernels) that reflect the structure of the data. Time-series data is challenging to design kernels for because of difficulties in reflecting the data structure (including time length). In this paper, a kernel design applicable to dynamical systems was required. Several methods were proposed, based on the subspace angle with kernel methods such as an auto-regressive moving average (ARMA) model [45]. We generalized to nonlinear dynamics without any specific underlying model, into which the Koopman spectrum of dynamics is incorporated. We called the kernels *Koopman spectral kernels*.

For calculating the similarity between the dynamical systems DS_*i*_ and DS_*j*_, we compute Koopman spectral kernels based on the idea of Binet-Cauchy kernels. In the unifying viewpoint [45], Binet-Cauchy kernels are a representation including various kernels [46–49], that serve two strategies. One is the trace kernel, which directly reflects the properties of the temporal evolution of the dynamical systems, including diffusion kernels [46] and graph kernels [47]. The second strategy is the determinant kernel, which extracts coefficients of dynamical systems, including the Martin distance [48] and the distance based on the subspace angle [49]. However, richer information about system trajectories does not necessarily increase the expressiveness for classifications with real-world data. Therefore, we also expanded the kernel of principal angle [50] to applications with Koopman spectral analysis, which is called the *Koopman kernel of principal angle*. The principal angle kernel is theoretically a simple case of the trace kernel [45], which is defined as the inner product of linear subspaces in this feature space. In our previous work, the Koopman kernel of principal angle showed the most effective expressiveness in spite of using only Koopman modes for the calculations [32]. In this paper, therefore, we compute the Koopman kernel of principal angle with the inner product of the Koopman modes, and leave the system trajectory and initial conditions aside.

The kernel of principal angle can be computed using the Koopman modes given by DMD with reproducing kernel. With respect to DS_*i*_, we define the kernel of principal angles as the inner product of the Koopman modes in the feature space: $A^{*}A={\hat{T}}_{i}^{-1}{{\bar{S}}_{i}}^{-1/2}{{\bar{B}}_{i}}^{*}{HG}_{yyii}H{\bar{B}}_{i}{{\bar{S}}_{i}}^{-1/2}{\hat{T}}_{i}$, where *i* is an index of the matrix generated by DMD with reproducing kernels applied to DS_*i*_. If the rank of $\hat{F}$ is *r*_*i*_, A^*^A is a *r*_*i*_-order square matrix. Also for DS_*j*_, we create a similar matrix B^*^B. Furthermore, we define the inner product of the linear subspaces between DS_*i*_ and DS_*j*_ as $A^{*}B={\hat{T}}_{i}^{-1}{{\bar{S}}_{i}}^{-1/2}{{\bar{B}}_{i}}^{*}{HG}_{yyij}H{\bar{B}}_{j}{{\bar{S}}_{j}}^{-1/2}{\hat{T}}_{j}$. *G*_*yyij*_ is a *n*_*i*_ × *n*_*j*_ matrix obtained by picking up the upper-right part of the centered Gram matrix obtained by connecting ***Y***_*i*_ and ***Y***_*j*_ in series (*n*_*i*_ and *n*_*j*_ are the lengths of the time series). Then, using these matrices, we solve the following generalized eigenvalue problem:

$$\left(\begin{array}{cc} 0 & {\left(A^{*}B\right)}^{*}\\ A^{*}B & 0 \end{array}\right)V=\lambda_{ij}\left(\begin{array}{cc} B^{*}B & 0\\ 0 & A^{*}A \end{array}\right)V,$$ (20)

where the size of ***λ***_*ij*_ is finally adjusted to *r*_ij_ = min(*r*_i_, *r*_j_) in descending order, and ***V*** is a generalized eigenvector. The eigenvalue ***λ***_*ij*_ is the kernel of principal angle. Note that for DMD without reproducing kernels, *A* and *B* can be calculated directly because these matrices are of finite dimensions.

### Embedding of the dynamics

For embedding of the distance matrix with our kernels, components of the distance matrix between dynamical systems in the feature space were obtained using *dist*(*DS*_*i*_,*DS*_*j*_) = *k*(***A***_*i*_,***A***_*i*_) + *k*(***A***_*j*_,***A***_*j*_) − 2*k*(***A***_*i*_,***A***_*j*_). We visualized by multidimensional scaling (MDS) with the distance matrix.

### Prediction of the probability of a successful shot

For predicting the outcome of a team’s attacking movement (i.e., shot), we computed the probability of the shot outcome because the shot outcome is probabilistic rather than deterministic. Thus, we used classifiers outputting the posterior probability such as a naive Bayes classifier. A naive Bayes classifier is a well-known and practical probabilistic classifier and has been employed in many applications. Since we had a relatively small dataset (192 attack-segment in total), we evaluated the median error rate, i.e., the rate of false negatives and false positives by testing at five times in analogous ways of 5-fold cross-validation. The results from applying other classifiers are shown in S3 Note and S11A and S11B Fig, respectively. The performances of other methods were inferior to that of the naive Bayes classifier.

## Supporting information

S1 Note Empirical examples of nonlinear oscillator system. Nonlinear oscillator system we used is described.
(DOCX)

S2 Note Additional results of DMD. Additional results of DMD for fish-schooling model simulation data are described.
(DOCX)

S3 Note Additional results of DMD with reproducing kernels. Additional results of DMD with reproducing kernels for basketball data are described.
(DOCX)

S1 Fig Distance matrices for fish-schooling model. Distance matrices (A) with fixed arrays among individuals and (B) of the nearest individuals at each time for fish-schooling model simulation data are shown.
(TIF)

S2 Fig Time series for fish-schooling model. Three types of distance matrices for three fish-schooling model simulation behavior are shown.
(TIF)

S3 Fig Spatiotemporal DMD mode without boundary condition. Configurations are the same as Fig 2 right (in boundary condition). The temporal DMD modes (A, D, G) are shown in the spectra as a function of temporal frequency. Using the results of the frequency spectra (A, D, G), we separated the spatial modes into low (0.5–1.5 Hz: B, E, H) and high (2–3 Hz: C, F, I) frequency domains. (G-I) vanished the frequency peak and DMD modes in parallel behavior.
(TIF)

S4 Fig DMD spectrum using fixed distance series and xy coordinates. Configurations are the same as Fig 2 right (using the nearest distance).
(TIF)

S5 Fig Reconstruction error in DMD using various input matrices. Reconstruction error in DMD using three types of distance matrices for three fish-schooling model simulation behavior are shown.
(TIF)

S6 Fig Transverse dynamic structure factor in the boundary condition. Configuration is the same as Fig 3 (longitudinal dynamic structure factor).
(TIF)

S7 Fig Dynamic structure factor without boundary. Configuration is the same as Fig 3 (in boundary condition).
(TIF)

S8 Fig Typical time series in basketball data. Three types of typical time series in basketball data we analyzed are shown.
(TIF)

S9 Fig Reconstruction error in DMD and that with reproducing kernel. The horizontal axis is the same as Fig 5 (classification error). The reconstruction error of the input matrices with respect to the Euclid distance and Cartesian coordinates for the original DMD were too large to plot.
(TIF)

S10 Fig Embedding of the DMD in the fish-schooling model. The rightmost (D) is the comparable result using the existing parameters specific in the biological group behavior.
(TIF)

S11 Fig Prediction results in two classifiers. Configuration is the same as Fig 5 (Naive Bayes classifier).
(TIF)

S12 Fig Embedding of the kernel of the DMD. Configurations are the same as Fig 4 right (DMD with reproducing kernel). The spectral kernel using only one distance was not be computed because of its low expressiveness.
(TIF)

S1 Table Values for each parameter in schooling model. Values for each parameter used in schooling model simulation are described.
(DOCX)

S1 Video Swarm behavior in schooling model. Time series are the same as S2 Fig.
(MP4)

S2 Video Torus behavior in schooling model. Time series are the same as S2 Fig.
(MP4)

S3 Video Parallel behavior in schooling model. Time series are the same as S2 Fig.
(MP4)

S4 Video Basketball attacking segment. Time series are the same as S8 Fig. Only four important distances are visualized (colors are same as Fig 4).
(MP4)

S5 Video A typical example of the true positive in score prediction. The prediction is performed based on the best classifier in this study. In this example, the attackers created the shot chance and made the shot successfully.
(MP4)

S6 Video A typical example of the true negative in score prediction. The prediction is performed based on the best classifier in this study. In this example, the defenders performed good defense and the attacker missed the shot.
(MP4)

S7 Video A typical example of the false positive in score prediction. The prediction is performed based on the best classifier in this study. In this example, the attackers created the chance but missed the shot.
(MP4)

S8 Video A typical example of the false negative in score prediction. The prediction is performed based on the best classifier in this study. In this example, the defenders performed good defense but the shot was successfully made.
(MP4)
