# Supplementary material for: Prediction and classification in equation-free collective motion dynamics
Source: PLoS Comput Biol. 2018 Nov 5;14(11):e1006545. doi: 10.1371/journal.pcbi.1006545 (PMC6237418; doi:10.1371/journal.pcbi.1006545)
Supplement: S1 Note — Nonlinear oscillator system we used is described. (DOCX) [file pcbi.1006545.s001.docx]

**Note S1. Empirical examples of nonlinear oscillator system**

To illustrate how temporal frequency and spatial domains can be decomposed in DMD (Fig. 1), we here consider a toy nonlinear oscillator system modeled by $f\left( x,t \right)=\mathrm{sech} \left( x+a \right)\exp\left( i\omega_{1}t \right)+\mathrm{sech} \left( x \right)\tanh(x)\exp\left( i\omega_{2}t \right)$, where$x$ is data dimension and $t$ is time. We set $a=0.3$, $\omega_{1}=2$, and $\omega_{2}=3$.

In this example of combinations of hyperbolic and trigonometric functions, DMD can achieve almost perfect decomposition in ideal cases [1]. When the data is translated or rotated, the decomposition into accurate modes may be difficult, but DMD can still reconstruct and reproduce the data [1].

**Reference**

1. Kutz JN, Brunton SL, Brunton BW, Proctor JL. Dynamic Mode Decomposition: Data-Driven Modeling of Complex Systems: SIAM; 2016.
