# Supplementary material for: Prediction and classification in equation-free collective motion dynamics
Source: PLoS Comput Biol. 2018 Nov 5;14(11):e1006545. doi: 10.1371/journal.pcbi.1006545 (PMC6237418; doi:10.1371/journal.pcbi.1006545)
Supplement: S2 Note — Additional results of DMD for fish-schooling model simulation data are described. (DOCX) [file pcbi.1006545.s002.docx]

**Note S2. Additional results of DMD**

**DMD results for different input matrices.** We input three types of matrices into DMD (rows are time series and columns are data dimension in all matrices). The first was a raw Cartesian coordinate time series, in which x and y coordinates for each fish-particle are arranged in a row. Second is a distance matrix with fixed arrays among individuals. The rows and columns in Fig. S1A represent the same agent indices even if the time changes. When making an input matrix, we choose combinations without duplication (i.e. 2016 combinations for 64 individuals) and arrange them in a row. The third is a sorted distance matrix of the nearest individuals at each time. Examples of the time series for the three input matrices are shown in Fig. S2.

The results for the temporal DMD modes exhibited a relatively wide and strong spectrum, a wide and weak spectrum, and a narrow but strong spectrum for the swarm, torus, and parallel behaviors, respectively (Figs 2A, D, G). For the spectrum of the parallel behavior, low frequency (0.5-1.5 Hz) peaks may indicate alignments and transient interactions resulting from a collision with the wall, because the parallel motion has the widest alignment area and additional simulations without a boundary wall had no low-frequency peak (Fig. S3).

As the temporal frequency spectra, the power spectra of the spatial DMD modes for the swarm (B-C), parallel (H-I), and torus behavior (2E-F) were stronger in this order. Furthermore, these spectra appeared more distinctly with the sorted nearest neighbor input matrix than did the spectra from the unsorted distance input matrix or the raw Cartesian input matrix (Fig. S4).

**DMD reconstruction error.** We calculated the error as the mean value of $\left( 1/\tau\right)\sum_{t=1}^{\tau-1} \left\| \boldsymbol{y}_{t+1}-\boldsymbol{\Phi}\boldsymbol{\Lambda}\boldsymbol{\Phi}^{\dagger}\boldsymbol{y}_{t} \right\|$ across data dimensions, which indicates the difference between the actual data at the next step and prediction data with the DMD. Results showed that among all behavior and input matrices, the reconstruction error was very small, under 10^-2^ m (Fig. S5). This suggests that the DMD reconstructions were successful.

**Embedding with distance matrix.** Figure S10 shows examples of the embedding of the DMD for the schooling model simulation, using multidimensional scaling (MDS) with the distance matrix, computed with the Koopman kernel of principal angle. Circle, triangle, and x indicate torus, parallel, and swarm behavior, respectively. For the sorted distance (Fig. S10B), the three types of behavior apparently separated. Using unsorted distance (Fig. S10A), the most of parallel behavior was correctly separated but the others did not. For positional information (Fig. 10C), none of the behavior types were distinguished. We additionally show the existing specific parameters such as polarization and angular momentum (the calculation is based on [2]) also performed clear discrimination (see Fig. S10D). Note that for the discrimination, the advantage of our method is that we do not need the prior knowledge about the labeled group behaviors. In this case, because of distinctness of the three behavioral shapes, both our method and the existing specific parameters show clear discrimination.
