# Supplementary material for: Prediction and classification in equation-free collective motion dynamics
Source: PLoS Comput Biol. 2018 Nov 5;14(11):e1006545. doi: 10.1371/journal.pcbi.1006545 (PMC6237418; doi:10.1371/journal.pcbi.1006545)
Supplement: S3 Note — Additional results of DMD with reproducing kernels for basketball data are described. (DOCX) [file pcbi.1006545.s003.docx]

**Note S3. Additional results of DMD with reproducing kernels**

**Reconstruction error estimation using pre-imaging.** Although DMD with reproducing kernels cannot directly extract a feature in the data space because of the decomposition in feature space, we estimated the difference between the original data and the reconstructed sequence in data space. To perform reconstruction error estimation in data space, we solved the pre-image problem [2], which is a reverse-mapping method from feature space back to input space. Although the inverse of the feature map typically does not exist, there is usually a simple relationship between the feature and input spaces. In this study, we use a non-iterative procedure with only linear algebra [2] as follows. (A) First, we computed the distance in the feature space. (B) Second, we transformed that into the distance in input space. (C) Third, we solved the pre-image problem. (D) Finally, we estimated the reconstruction error.

First, we computed (A) the distance in the feature space.

| ${{{\tilde{\boldsymbol{d}}}_{t+1}}^{2}=\left\Vert\phi_{\boldsymbol{x}_{t+1}}-\hat{\phi}_{\boldsymbol{x}_{t+1}} \right\Vert}^{2}$ $={\phi_{\boldsymbol{x}_{t+1}}}^{T}\phi_{\boldsymbol{x}_{t+1}}+{\hat{\phi}_{\boldsymbol{x}_{t+1}}}^{T}\hat{\phi}_{\boldsymbol{x}_{t+1}}-2{\phi_{\boldsymbol{x}_{t+1}}}^{T}\hat{\phi}_{\boldsymbol{x}_{t+1}},$ |  |
| --- | --- |

where$\phi_{\boldsymbol{x}_{t+1}}$ and $\hat{\phi}_{\boldsymbol{x}_{t+1}}$ are the actual and reconstructed feature maps at the next step, respectively. Note that these are of infinite dimensions, and thus we cannot directly compute$\phi_{\boldsymbol{x}_{t+1}}$ and $\hat{\phi}_{\boldsymbol{x}_{t+1}}$ but can compute the above terms in Eq. (1) as a product representation. The first term can be easily computed using ${{\phi_{\boldsymbol{x}_{t+1}}}^{T}\phi_{\boldsymbol{x}_{t+1}}=\mathcal{M}_{2}^{*}\mathcal{M}}_{2}$, where $\mathcal{M}_{2}:=\left[ \phi_{\boldsymbol{x}_{2}},..,\phi_{\boldsymbol{x}_{\tau}} \right]$. For the second term, we briefly express the component of $\hat{\phi}\left( \boldsymbol{x}_{t+1} \right)$ as:

| $\hat{\phi}\left( \boldsymbol{x}_{t+1} \right)=\sum_{j=1}^{p} \lambda_{j}\varphi_{j}\left( \boldsymbol{x}_{t} \right)\bar{\varphi}_{j}$ $=\sum_{j=1}^{p} \lambda_{j}\boldsymbol{a}^{\boldsymbol{*}}\bar{S}^{1/2}\bar{B}^{\boldsymbol{*}}\mathbf{H}\mathcal{M}_{1}^{*}\phi_{\boldsymbol{x}_{t}}\mathcal{M}_{1}\mathbf{H}\bar{B}\bar{S}^{-1/2}\boldsymbol{b}_{j}$ $=\mathcal{M}_{1}\boldsymbol{v}_{t+1},$ |  |
| --- | --- |

where $\boldsymbol{v}_{t+1}=\sum_{j=1}^{p} c_{j}\mathbf{H}\bar{B}\bar{S}^{-1/2}\boldsymbol{b}_{j}$ is the finite-dimensional vector and $c_{j}={\lambda_{j}\boldsymbol{a}}^{\boldsymbol{*}}\bar{S}^{1/2}\bar{B}^{\boldsymbol{*}}\mathbf{H}\mathcal{M}_{1}^{*}\phi_{\boldsymbol{x}_{t}}$ is a scalar value (because $\mathcal{M}_{1}^{*}\phi_{\boldsymbol{x}_{t}}$ is the part of $G_{yy}$ and can be computed). Then, we can obtain the second term in Eq. (11):

| ${\hat{\phi}_{\boldsymbol{x}_{t+1}}}^{T}\hat{\phi}_{\boldsymbol{x}_{t+1}}={\boldsymbol{v}_{j}^{*}\mathcal{M}_{1}^{*}\mathcal{M}}_{1}\boldsymbol{v}_{j},$ |  |
| --- | --- |

where ${\mathcal{M}_{\tau}^{*}\mathcal{M}}_{\tau}=G_{yy}$ is computable. Similarly, the third term in Eq. (11) can be obtained:

| ${\phi_{\boldsymbol{x}_{t+1}}}^{T}\hat{\phi}_{\boldsymbol{x}_{t+1}}={{\phi_{\boldsymbol{x}_{t+1}}}^{*}\mathcal{M}}_{1}\boldsymbol{v}_{j},$ |  |
| --- | --- |

where ${{\phi_{\boldsymbol{x}_{t+1}}}^{*}\mathcal{M}}_{1}$ is a part of $G_{yy'}^{*}={{\mathcal{M}_{2}}^{*}\mathcal{M}}_{1}$ and can be computed. In practice, the correction of these terms will be needed because of the centralization caused by DMD with reproducing kernels.

Second, we transformed it into (B) the distance into input space:

| $d^{2}=-2{\sigma^{'}}^{2}\log\left( 1-\frac{\tilde{d}^{2}}{2} \right).$ |  |
| --- | --- |

Third, we solved (C) the pre-image problem under the constraints of the above input distance. For the *n* neighbors $\boldsymbol{X}_{n}=[\boldsymbol{x}_{1},\ldots,\boldsymbol{x}_{n}]$ in the input data matrix (in this study, we set *n* = 20), we ﬁrst center them as ${\bar{\boldsymbol{X}}}_{n}$ at their centroid $\bar{\boldsymbol{x}}=\left( 1/n \right)\sum_{i=1}^{n} \boldsymbol{x}_{i}$ (i.e., the column sums of ${\bar{\boldsymbol{X}}}_{n}$ are zero). Assuming that the training patterns span a *q*-dimensional space (i.e., $\boldsymbol{X}_{n}$is of rank *q*), we obtain the singular value decomposition of ${\bar{\boldsymbol{X}}}_{n}$ as

| ${\bar{\boldsymbol{X}}}_{n}=\boldsymbol{U\Lambda}\mathbf{V}^{*}=\mathbf{UZ,}$ |  |
| --- | --- |

where $\mathbf{U=}\left[ \boldsymbol{e}_{1}\boldsymbol{,}\ldots,\boldsymbol{e}_{q} \right]$ is a $d\times q$ matrix with orthonormal columns $\boldsymbol{e}_{i}$ and $\mathbf{Z=}\left[ \boldsymbol{z}_{1}\boldsymbol{,}\ldots,\boldsymbol{z}_{n} \right]$ is a $q\times n$ matrix with columns $\boldsymbol{z}_{i}$being the projections of $\boldsymbol{x}_{i}$ onto the $\boldsymbol{e}_{i}$’s. Besides, the squared distance of $\boldsymbol{x}_{i}$to the origin, which is still at the centroid, is equal to $\left\| \boldsymbol{z}_{i} \right\|^{2}$. Then, we collect these distances into an *n*-dimensional vector as $\boldsymbol{d}_{0}^{2}=\left[ \left\| \boldsymbol{z}_{1} \right\|^{2},\ldots,\left\| \boldsymbol{z}_{n} \right\|^{2} \right]^{'}$ and $\boldsymbol{d}^{2}=\left[ d_{1}^{2},\ldots,d_{n}^{2} \right]^{'}$. Previous work [2] assumed that the required pre-image $\hat{\boldsymbol{x}}$ is in the span of the *n* neighbors and settled for the least-square solution $\hat{\boldsymbol{z}}$. Then, the pre-image can be obtained as

| $\hat{\boldsymbol{z}}=-\frac{1}{2}\boldsymbol{\Lambda}^{-1}\mathbf{V}^{*}\left( \boldsymbol{d}^{2}-\boldsymbol{d}_{0}^{2} \right).$ |  |
| --- | --- |

For transforming back to the original coordinate system in the input space, we have

| $\hat{\boldsymbol{x}}=\mathbf{U}\hat{\boldsymbol{x}}\boldsymbol{+}\bar{\boldsymbol{x}}.$ |  |
| --- | --- |

This result shows that the all the reconstruction performances of the DMD with reproducing kernels outperformed those of the original DMD (Fig. S9). The reconstruction error of the input matrices with respect to the Euclid distance and Cartesian coordinates for the original DMD were too large to plot.

**Embedding with distance matrix.** Figure 4 right shows embedding via MDS with the distance matrix of the Koopman spectral kernels for basketball data contoured by frequencies of success and failure of the shot. The kernel of DMD showed poor distribution and poor expressiveness (Fig. S12).

**Prediction of the probability of a successful shot.** For the basketball data, Fig. 5 shows the result of applying the naive Bayes classifier. The Koopman kernel principal angles derived by inputting four relevant distances demonstrated the minimum error of 35.9%. The results from applying the K-nearest neighbor method and the relevance vector machine (RVM) classifiers are shown in Fig. S11A and S11B, respectively. The performance of the RVM and the K-nearest neighbor methods were inferior to that of the naive Bayes classifier.

**Reference**

2. Kwok J-Y, Tsang I-H. The pre-image problem in kernel methods. IEEE transactions on neural networks. 2004;15(6):1517-25.
