# Supplementary figures and images for: Prediction and classification in equation-free collective motion dynamics
Source: PLoS Comput Biol. 2018 Nov 5;14(11):e1006545. doi: 10.1371/journal.pcbi.1006545 (PMC6237418; doi:10.1371/journal.pcbi.1006545)

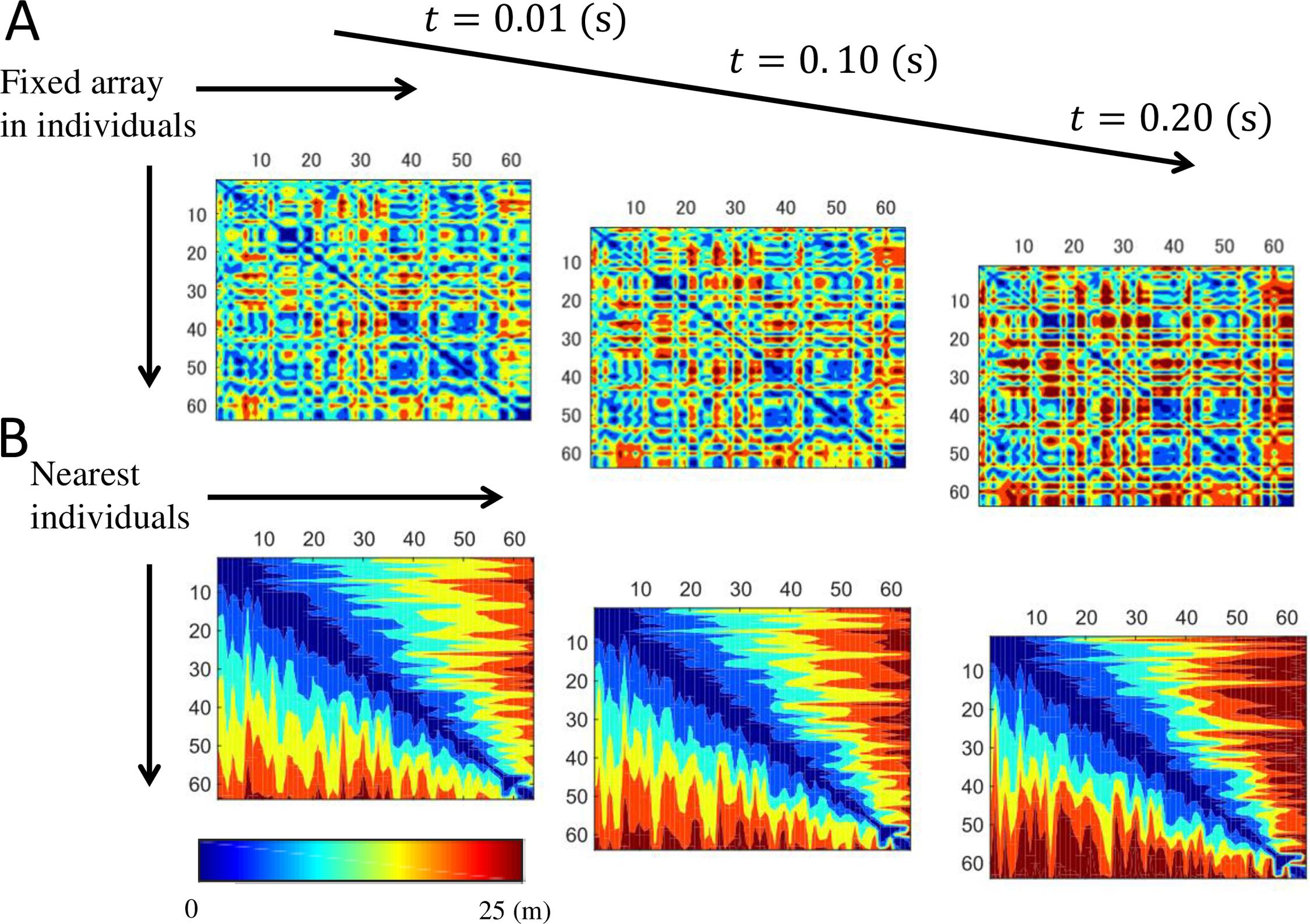

Supplement: S1 Fig — Distance matrices (A) with fixed arrays among individuals and (B) of the nearest individuals at each time for fish-schooling model simulation data are shown. (TIF) [file pcbi.1006545.s004.tif]

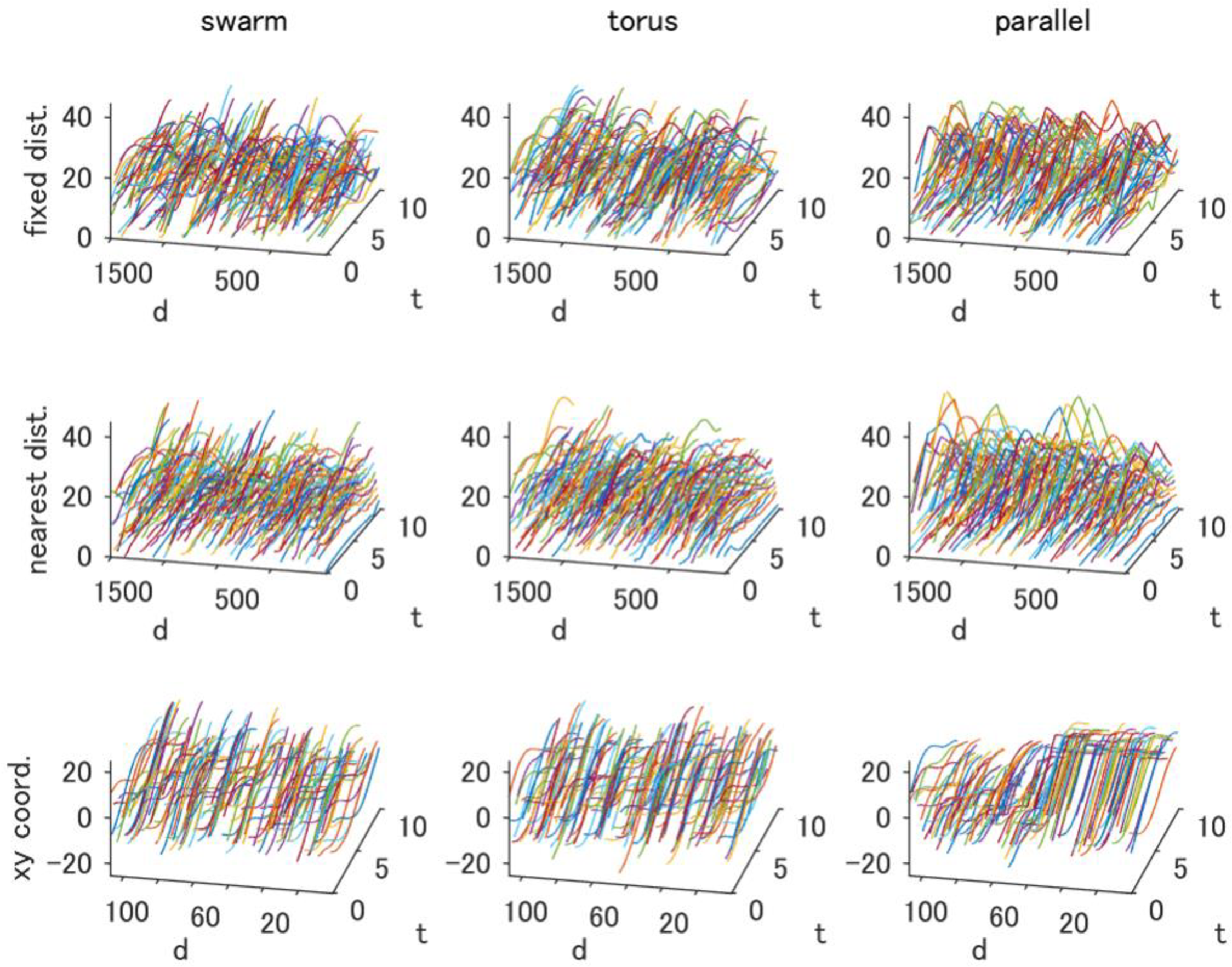

Supplement: S2 Fig — Three types of distance matrices for three fish-schooling model simulation behavior are shown. (TIF) [file pcbi.1006545.s005.tif]

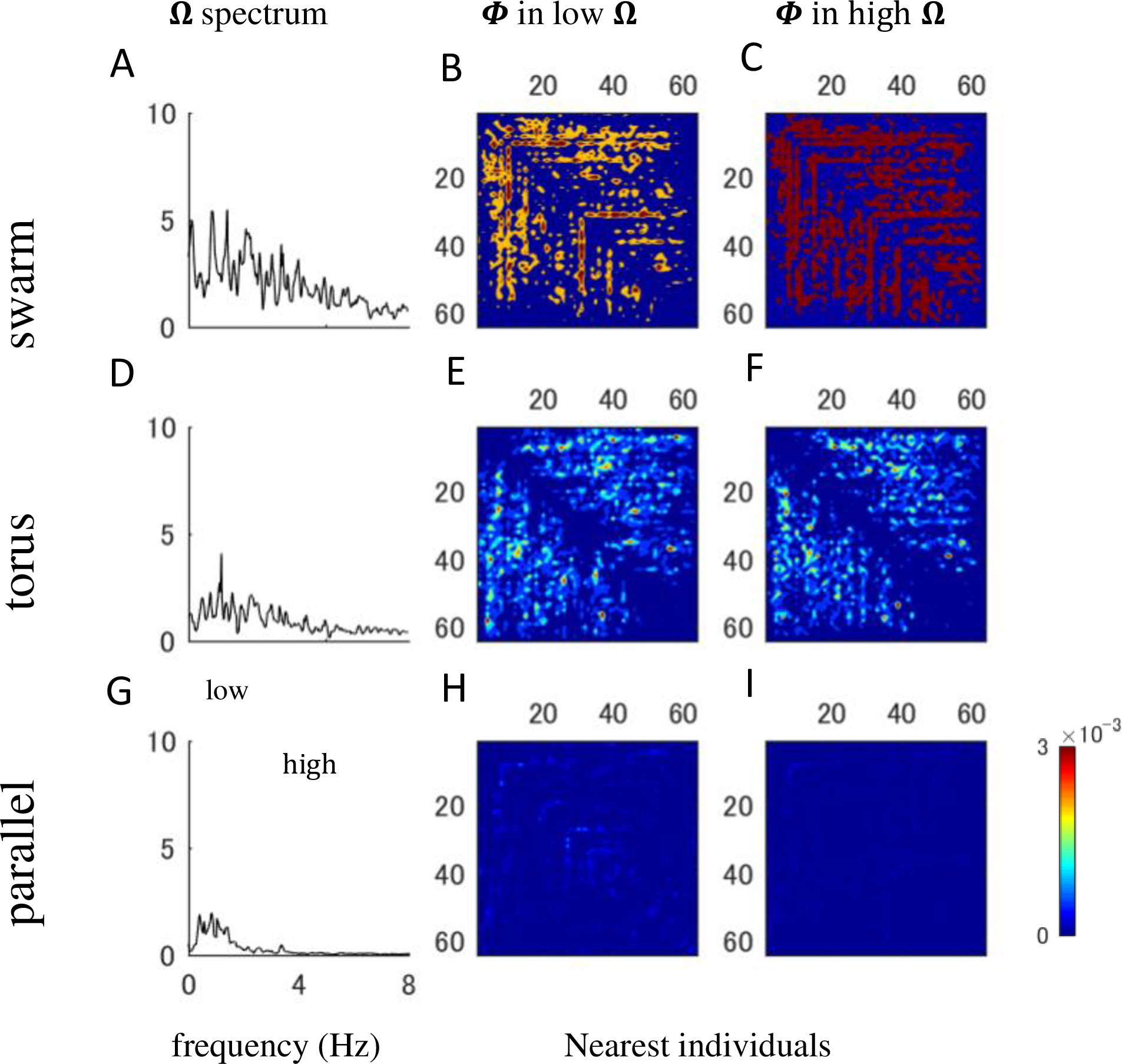

Supplement: S3 Fig — Configurations are the same as Fig 2 right (in boundary condition). The temporal DMD modes (A, D, G) are shown in the spectra as a function of temporal frequency. Using the results of the frequency spectra (A, D, G), we separated the spatial modes into low (0.5–1.5 Hz: B, E, H) and high (2–3 Hz: C, F, I) frequency domains. (G-I) vanished the frequency peak and DMD modes in parallel behavior. (TIF) [file pcbi.1006545.s006.tif]

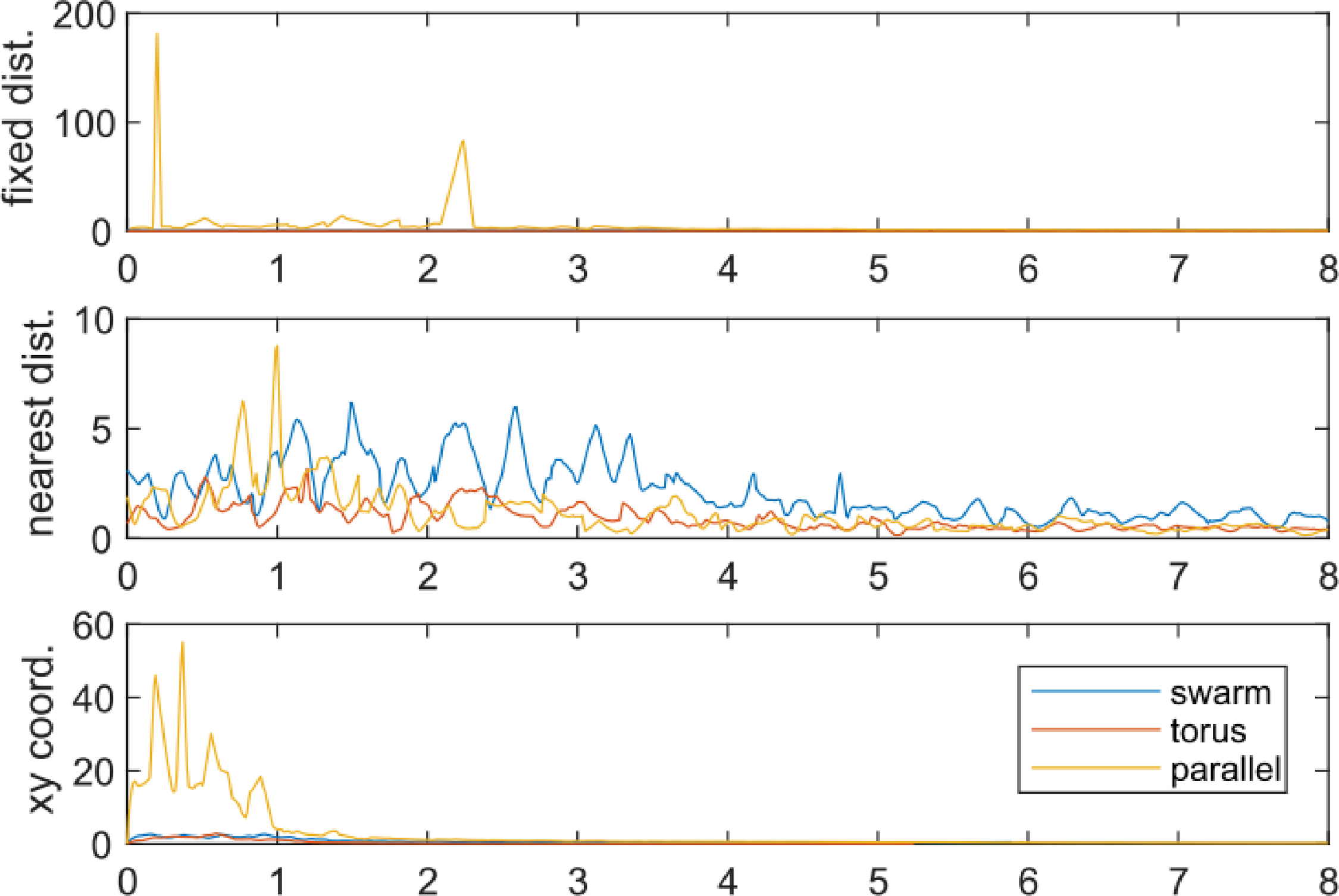

Supplement: S4 Fig — Configurations are the same as Fig 2 right (using the nearest distance). (TIF) [file pcbi.1006545.s007.tif]

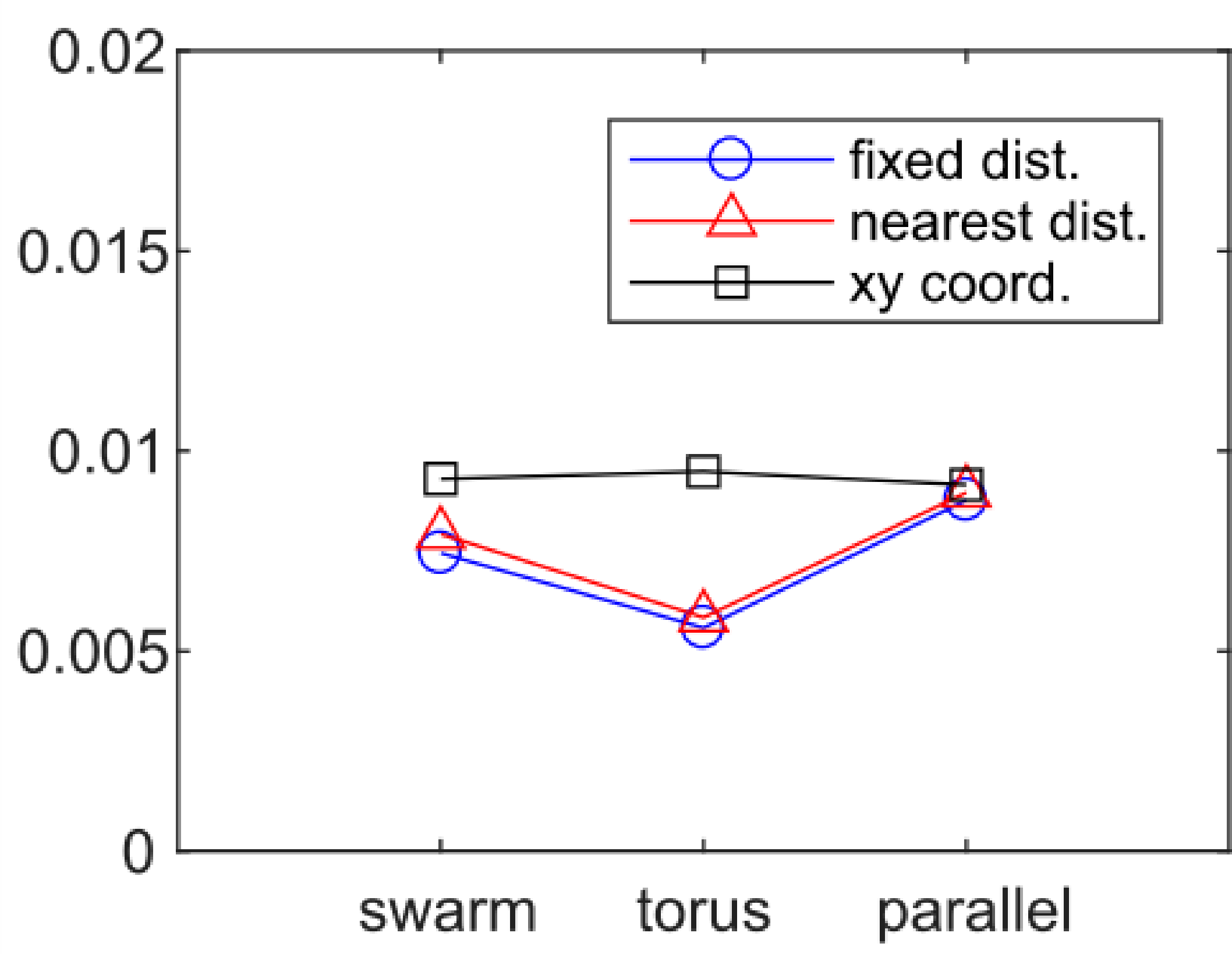

Supplement: S5 Fig — Reconstruction error in DMD using three types of distance matrices for three fish-schooling model simulation behavior are shown. (TIF) [file pcbi.1006545.s008.tif]

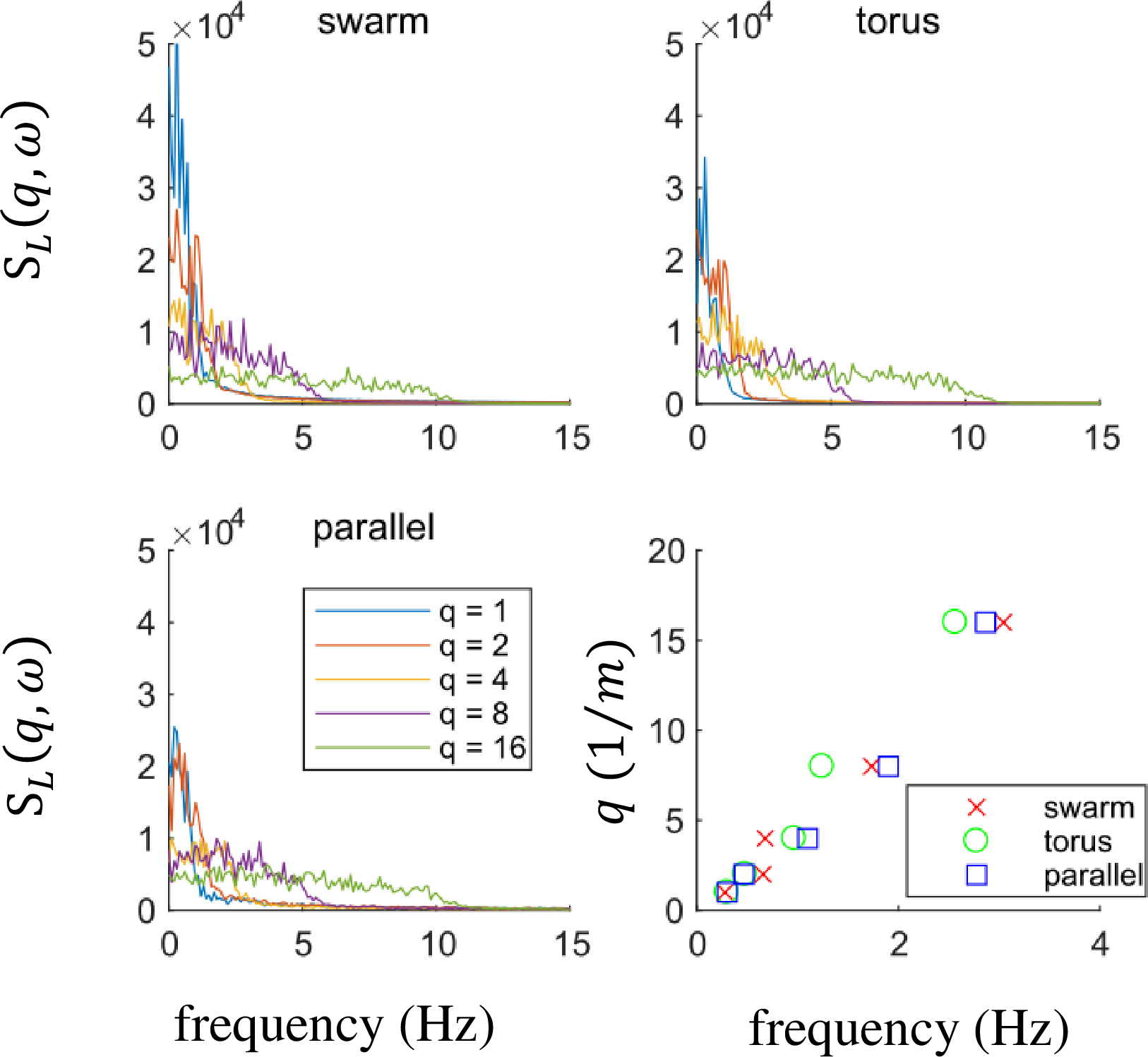

Supplement: S6 Fig — Configuration is the same as Fig 3 (longitudinal dynamic structure factor). (TIF) [file pcbi.1006545.s009.tif]

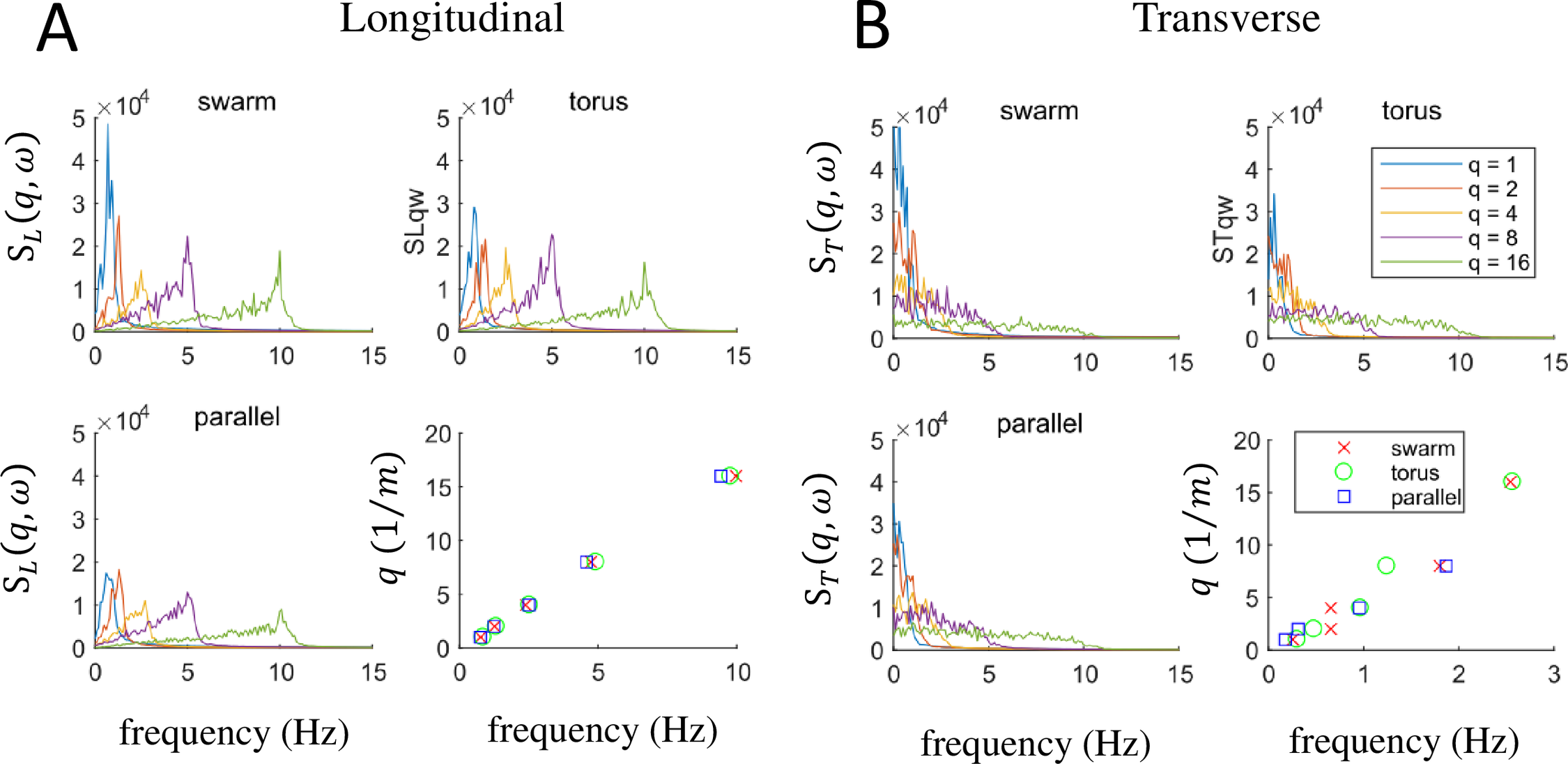

Supplement: S7 Fig — Configuration is the same as Fig 3 (in boundary condition). (TIF) [file pcbi.1006545.s010.tif]

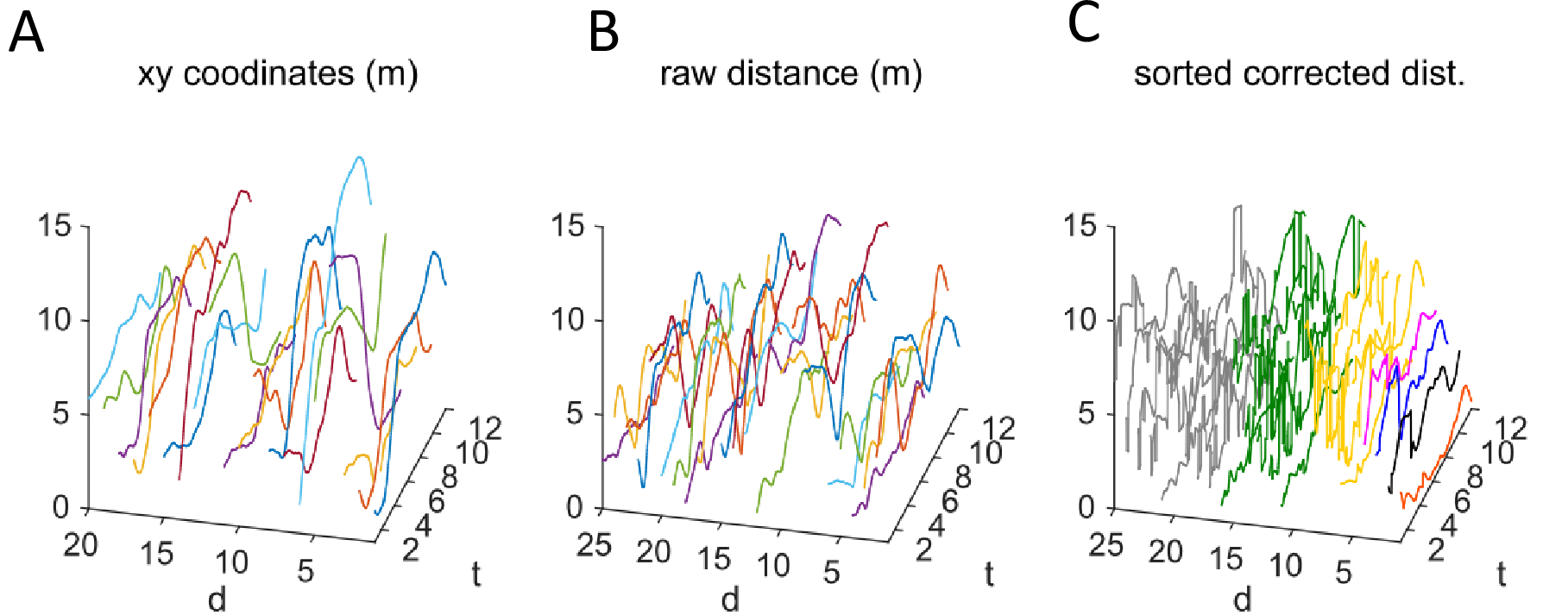

Supplement: S8 Fig — Three types of typical time series in basketball data we analyzed are shown. (TIF) [file pcbi.1006545.s011.tif]

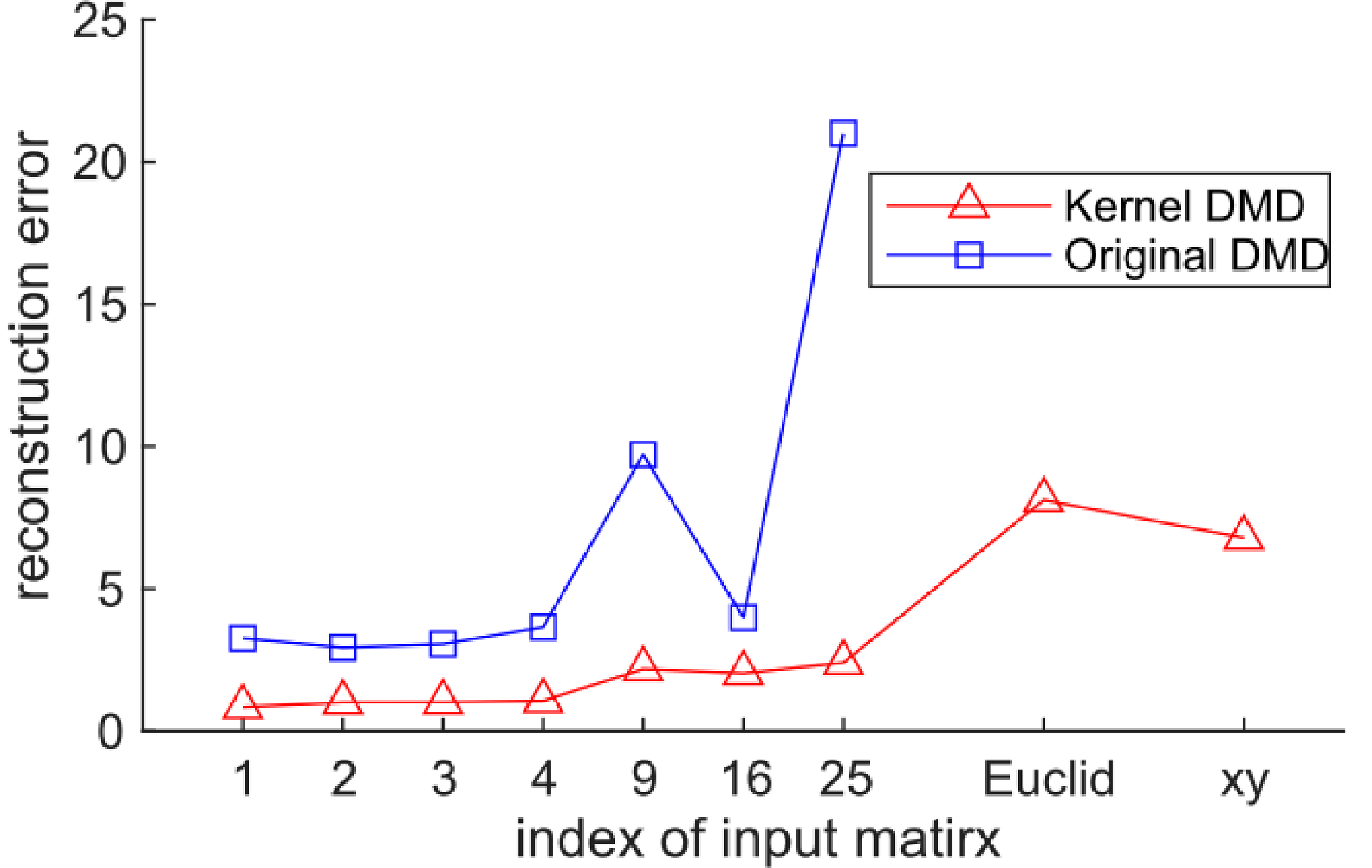

Supplement: S9 Fig — The horizontal axis is the same as Fig 5 (classification error). The reconstruction error of the input matrices with respect to the Euclid distance and Cartesian coordinates for the original DMD were too large to plot. (TIF) [file pcbi.1006545.s012.tif]

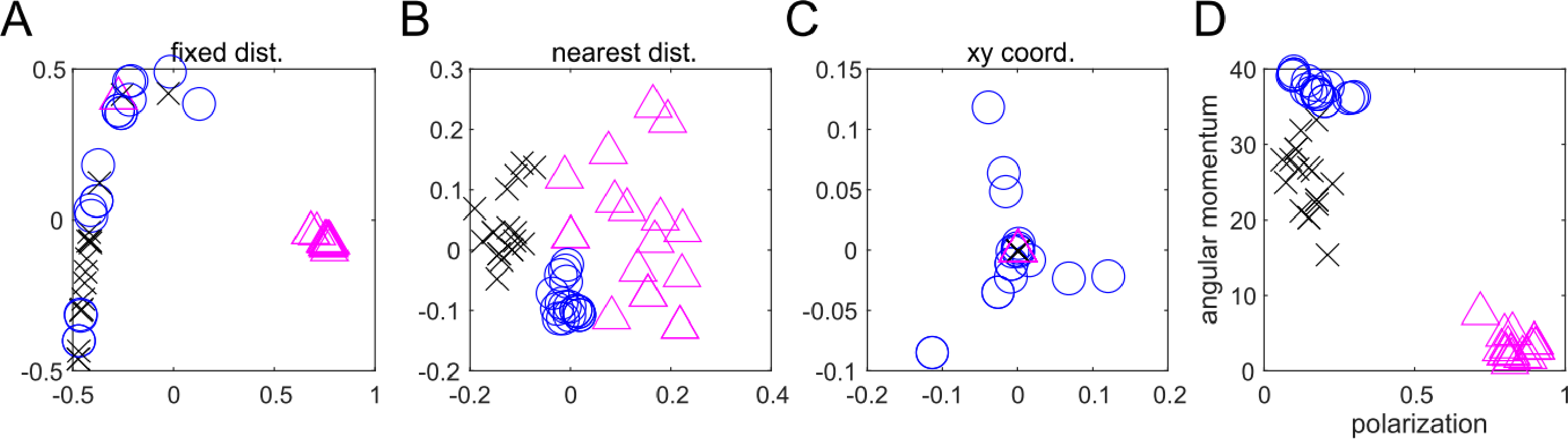

Supplement: S10 Fig — The rightmost (D) is the comparable result using the existing parameters specific in the biological group behavior. (TIF) [file pcbi.1006545.s013.tif]

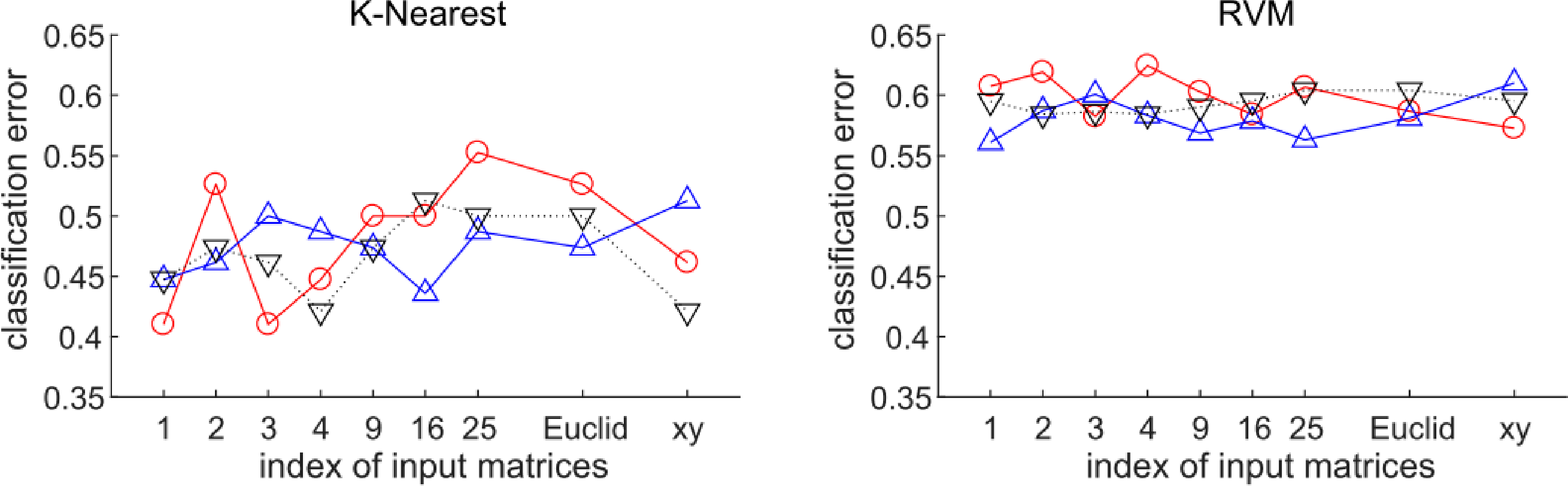

Supplement: S11 Fig — Configuration is the same as Fig 5 (Naive Bayes classifier). (TIF) [file pcbi.1006545.s014.tif]

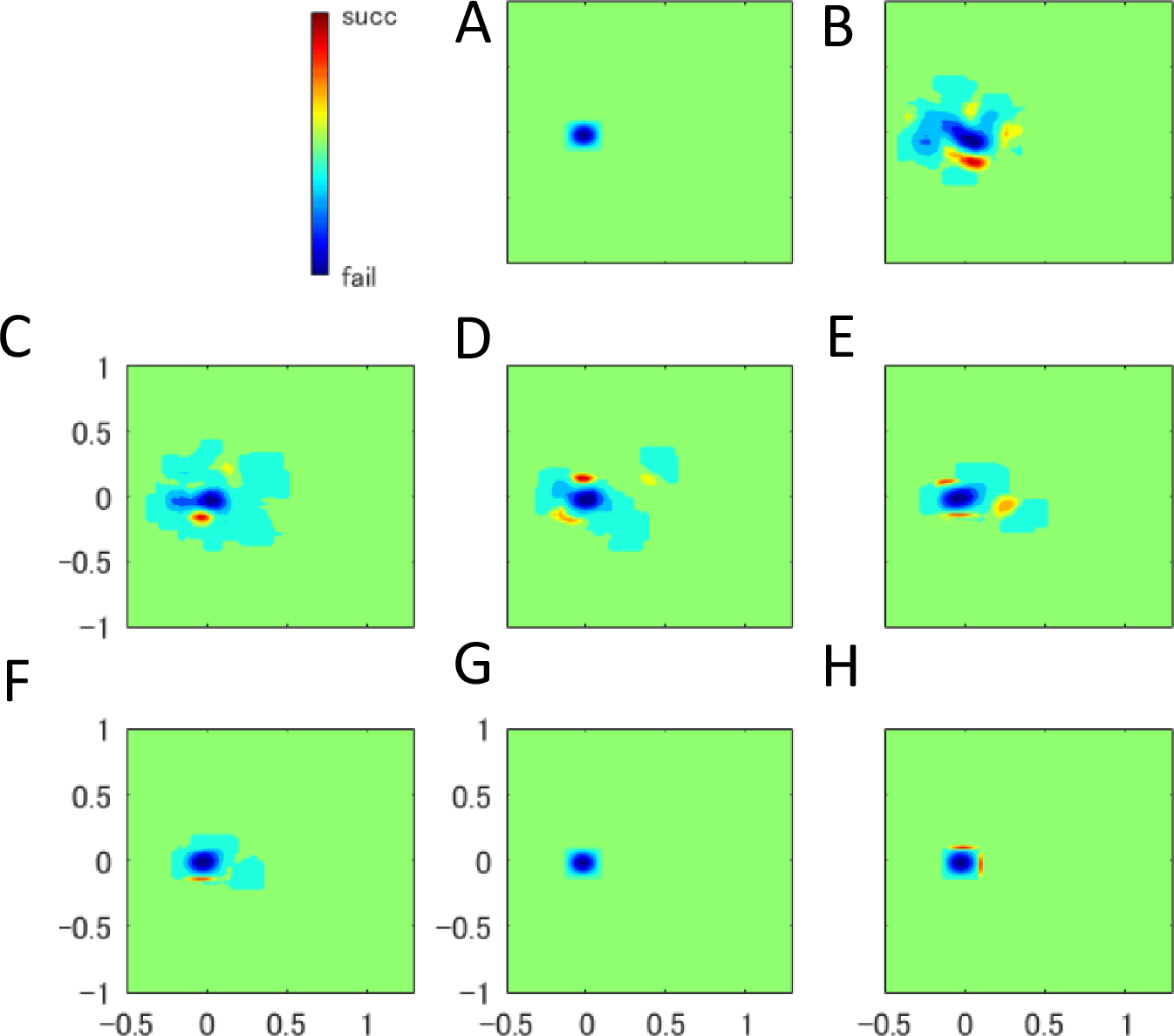

Supplement: S12 Fig — Configurations are the same as Fig 4 right (DMD with reproducing kernel). The spectral kernel using only one distance was not be computed because of its low expressiveness. (TIF) [file pcbi.1006545.s015.tif]
