# Supplementary material for: Prediction and classification in equation-free collective motion dynamics
Source: PLoS Comput Biol. 2018 Nov 5;14(11):e1006545. doi: 10.1371/journal.pcbi.1006545 (PMC6237418; doi:10.1371/journal.pcbi.1006545)
Supplement: S1 Table — Values for each parameter used in schooling model simulation are described. (DOCX) [file pcbi.1006545.s016.docx]

**3. Supplementary Table**

**Table S1. Values for each parameter in schooling model.**

| **Parameter** | **Values** |
| --- | --- |
| Population size *N* | 64 |
| Repulsion radius $r_{r}$ | 1 [m] |
| Orientation radius $r_{o}$ | 2,10,13 [m] |
| Attraction radius $r_{a}$ | 15 [m] |
| Maximum turning angle $\beta$ | 30 [deg] |
| Speed $\left\vert\vec{v_{i}} \right\vert$ | 4 [m/s] |
| Standard deviation $\sigma$ | 0.05 |
| Radius of boundary circle | 25, $\infty$ [m] |
